# Authentication of Linderae Radix through plant metabolomics coupled with a machine learning-enhanced *in situ* hyperspectral imaging approach

**DOI:** 10.1016/j.jpha.2025.101476

**Published:** 2025-10-28

**Authors:** Yangbin Lv, Hongwei Sun, Qiaoling Ding, Bangxu Chen, Hongwei Ye, Ning Xu, Chu Chu

**Affiliations:** a College of Pharmaceutical Science, Zhejiang University of Technology, Huzhou, Zhejiang, 313200, China; b Zhejiang Provincial Key Laboratory of TCM for Innovative R&D and Digital Intelligent Manufacturing of TCM Great Health Products, Huzhou, Zhejiang, 313200, China

**Keywords:** *L. aggregata*, Different root types, Plant metabolomics, Hyperspectral imaging, Machine learning algorithms, Visual *in situ* assessment

## Abstract

Linderae Radix, a medicinally significant herb with a history of over 2000 years, is highly esteemed for its potential to promote longevity. Derived from the tuberous roots of *Lindera aggregata* (*L. aggregata*), it encounters difficulties in being distinguished from non-medicinal parts, such as non-fusiform taproots and old roots in the herbal drug market. To address the problem, this study developed a new strategy that integrates non-targeted plant metabolomics with a machine learning-enhanced hyperspectral imaging (HSI) approach for *in situ* quality assessment. Firstly, a comprehensive metabolomics analysis was conducted using ultra-performance liquid chromatography-quadrupole time-of-flight mass spectrometry (UPLC-QTOF-MS) and gas chromatography-mass spectrometry (GC-MS) to identify 25 and 48 differential metabolites, respectively. Then, combined with machine learning algorithms, HSI in the 400–1000 nm band achieved visual *in situ* assessment of different types of *L. aggregata* roots. Second derivative (2^nd^D)-Savitzky-Golay (SG) smoothing-logistic regression (LR) models achieved 93.33% accuracy of the test set in spectral classification. Moreover, spectral pre-processing and characteristic wavelength selection led to high prediction accuracies for the content of significant components in *L. aggregata* using standard normal variate (SNV)-competitive adaptive reweighted sampling (CARS)-least squares support vector machine (LSSVM) and SNV-CARS-extreme learning machine (ELM) ($R_{P}^{2}$ > 0.87 for the test set). This is the first study to provide a visual representation of the content of marker compounds in *L. aggregata* roots, offering a rapid, non-destructive method for assessing the quality of Linderae Radix. It scientifically justifies the medicinal use of tuberous roots and illuminates rapid quality evaluation through morphological identification.

## Highlights

• A rapid, non-destructive method for assessing the quality of Linderae Radix.
• Integrated metabolomics, HSI, and machine learning for rapid quality assessment.
• Machine learning for establishing classification and quantitative prediction models**.**
• Visualize the spatial distribution of the norisoboldine content in drug slices.

## Graphical abstract

**Figure:**
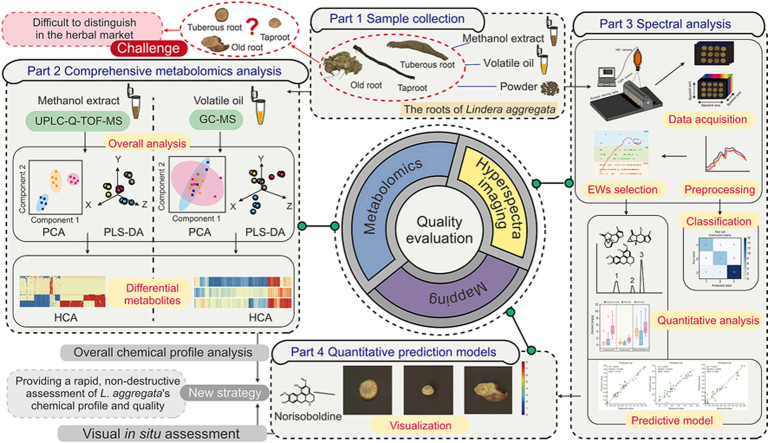

## 1 Introduction

Linderae Radix, also known as “Wuyao”, is derived from the tuberous root of *Lindera aggregata* (Sims) Kosterm. (*L. aggregata*), a traditional Chinese medicine with a rich history spanning over 2000 years. It is renowned for its therapeutic properties, which include promoting qi circulation, relieving pain, warming the kidneys, and dispelling cold, and has been a mainstay in clinical treatments for millennia [1]. It is also recognized for its potential to extend life as a tonic and for its effectiveness in alleviating dysmenorrhea, abdominal pain, indigestion, and frequent urination [2]. Despite its significant commercial demand, there has been a notable decline in the quality and efficacy of Linderae Radix in recent years, primarily due to post-processing morphological homogenization, which enables market adulteration with taproots and old roots that are more readily available [3]. The reason for the adulteration phenomenon is that the cultivation of *L. aggregata* is a lengthy process, with roots taking 8–10 years to mature for harvest, and the yield of tuberous roots is considerably lower than that of taproots [4]. The morphological differences among the three root types are significant, and their decoction slices are nearly indistinguishable, posing quality control challenges in the production of traditional Chinese medicines.

Several studies have compared the chemical and pharmacological differences among taproots, tuberous roots, and old roots of *L. aggregata* using various methods, including microscope image recognition, high-performance liquid chromatography (HPLC), near-infrared spectroscopy (NIRS), and hyperspectral imaging (HSI) [[3], [4], [5], [6], [7], [8]]. However, these methods face limitations in accuracy, efficiency, or convenience for sample classification. HPLC, a chemical analysis method, is time-consuming and destructive, and it often fails to reveal the complete chemical profile, including volatile components, among different root types of *L. aggregata*. Meanwhile, NIRS and HSI, though non-destructive, struggle to provide detailed chemical composition information due to the complexity of spectra and the need for chemometric analysis. This is particularly challenging for samples with highly similar morphology and composition, making it difficult to distinguish closely related herbs. Therefore, developing a rapid and efficient strategy to reveal the connection between chemical constituents and hyperspectral images is crucial for achieving “quality evaluation through morphological identification” of Linderae Radix samples in the market.

Non-targeted plant metabolomics, leveraging high performance liquid chromatography-mass spectrometry (HPLC-MS) and gas chromatography-mass spectrometry (GC-MS), is a standard method for probing the diverse array of non-volatile and volatile metabolites in biological samples [9]. This approach is ideal for the quality control of plant-derived natural medicines, as it enables the comprehensive detection of small molecule metabolites, discerns sample differences across various classifications—including origin [10], plant parts [7], and species [11]—and facilitates both qualitative and quantitative analyses. While the differences in isoquinoline alkaloid metabolites in tuberous roots and taproots of *L. aggregata* have been investigated, a comprehensive metabolomics study encompassing other key components, such as sesquiterpenes and volatile oils, across different root types is lacking [7,12]. Given the complexity of *L. aggregata*, an integrated non-targeted metabolomics strategy using HPLC-MS and GC-MS is essential for a complete understanding of its chemical variations.

HSI has become a prominent, non-polluting, rapid, and non-destructive technique, merging conventional imaging with spectral analysis to extract spatial and spectral data from samples [13]. With the advancement of machine learning, HSI integrated with machine learning has been extensively applied in the research, detection, quantification, and identification of food and natural medicinal plants, including quality assessment [14], origin classification [15], and detection of active ingredient content [16]. Although HPLC-based analyses and NIRS fingerprinting of *L. aggregata* roots with different morphologies have been reported, and a portable short-wave infrared microscopic hyperspectral imager combined with machine learning algorithms has been developed for classifying the geographic origin and root types of *L. aggregata* [6,7], no studies have yet integrated HSI spectral information with quantitative data on the main components of *L. aggregata* to construct quantitative prediction models.

**Fig. 1:**
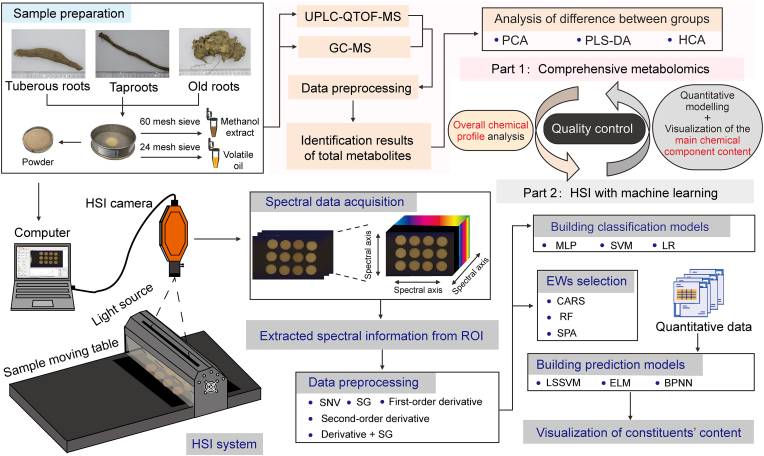
Schematic representation of comprehensive metabolomics and the proposed machine learning hyperspectral imaging method. UPLC-QTOF-MS: ultra-performance liquid chromatography-quadrupole time-of-flight mass spectrometry; GC-MS: gas chromatography-mass spectrometry; PCA: principal component analysis; PLS-DA: partial least squares discrimination analysis; HCA: hierarchical cluster analysis; HSI: hyperspectral imaging; ROI: region of interest; SNV: standard normal variate; SG: Savitzky-Golay; MLP: multi-layer perceptron; SVM: support vector machine; LR: logistic regression; EWs: effective wavelengths; CARS: competitive adaptive reweighted sampling; RF: random frog; SPA: successive projections algorithm; LSSVM: least squares support vector machine; ELM: extreme learning machine; BPNN: backpropagation neural network.

This study, therefore, combines comprehensive metabolomics data, quantitative constituent information, and visible (VIS)-near-infrared (NIR) hyperspectral data, which are enhanced by machine learning to elucidate the differences in chemical composition and indicator chemical constituents among *L. aggregata* samples of varying root types, including alcoholic extracts, volatile oils, powders, and decoction pieces. The schematic representation of comprehensive metabolomics and the proposed machine learning HSI method are shown in Fig. 1. Initially, ultra-performance liquid chromatography-quadrupole time-of-flight mass spectrometry (UPLC-QTOF-MS) and GC-MS metabolomics were employed to dissect the chemical profiles of tuberous roots, taproots, and old roots of *L. aggregata.* HSI was then utilized to gather spectral data from powdered samples of different root types, enabling classification and prediction of the content of three major differential volatile and non-volatile components: norisoboldine, linderane, and lindenenol. After pre-processing the spectra and selecting the characteristic wavelengths using machine learning algorithms, a quantitative prediction model with high accuracy was constructed. Finally, the reflectance of each pixel in the spectral image was extracted using a pixel mapping method to predict the distribution of norisoboldine content in *L. aggregata* with different root types.

## 2 Materials and methods

### 2.1 Chemicals and reagents

All chemicals utilized were of analytical reagent grade or superior. LC-MS grade formic acid was sourced from Tedia Company Inc. (Cincinnati, OH, USA). HPLC-grade methanol and acetonitrile were procured from Merck (Darmstadt, Germany). Analytical-grade formic acid and methanol were acquired from Tianjin Yongda Chemical Reagent Co., Ltd. (Tianjin, China) and Shanghai Titan Scientific Co., Ltd. (Shanghai, China), respectively. Norisoboldine, linderane, and lindenenol were obtained from Chengdu Must Biological Technology Co., Ltd. (Chengdu, China). Pure water was supplied by Hangzhou Wahaha Group Co., Ltd. (Hangzhou, China).

### 2.2 Plant materials

A total of 49 batches of *L. aggregata* samples were collected from wild picking and the drug control institute, tuberous roots (*n* = 29), taproots (*n* = 10), and old roots (*n* = 10) (*n* indicates the number of independent samples). All the samples were identified by Prof. Xin Peng (Ningbo Research Institute of Traditional Chinese Medicine), and the specimens were deposited at Zhejiang University of Technology (Table S1). Fresh *L.* *aggregata* samples were divided into tuberous roots, old roots, and taproots based on their distinct types. They are then washed and dried to a constant weight at 65 °C. The samples are stored in a desiccator for preservation.

### 2.3 UPLC-QTOF-MS-based untargeted metabolomics analysis

#### 2.3.1 Sample preparation

The dried *L.* *aggregata* sample was first crushed into a fine powder using a medicinal material crusher, then passed through a 60-mesh sieve, and stored in a glass dryer. Subsequently, 0.5 g of the powdered roots of *L. aggregata* were suspended in 5 mL of methanol. This suspension was subjected to ultrasonic extraction at 60 °C for 30 min. After extraction, the supernatant was filtered through a 0.45 μm filter membrane to remove particulates. The solvent was then evaporated under reduced pressure at 40 °C using a Genevac EZ-2 vacuum centrifugal concentrator (British Genevac company, Milton Keynes, UK). The resulting extracts were stored at 4 °C for subsequent analysis.

#### 2.3.2 UPLC-QTOF-MS analysis

*L. aggregata* root extracts were dissolved in methanol and filtered through a 0.22 μm membrane for UPLC-QTOF-MS analysis. The Agilent 1290 series UPLC system (Agilent Technologies, Santa Clara, CA, USA) was used for chromatography on a Poroshell 120 EC C_18_ column (4.6 mm × 100 mm, 2.7 μm, Agilent Technologies, Santa Clara, CA, USA) at 30 °C, with a gradient elution of 0.1 % formic acid in water (solvent A) and acetonitrile (solvent B): 0–10 min, 10%–20% B; 10–15 min, 20%–40% B; 15–17 min, 40%–90% B; 17–25 min, 10% B. The flow rate was 1 mL/min, and the injection volume was 5 μL. Detection was performed on an Agilent 6545 Q-TOF MS with ESI (Agilent Technologies, Santa Clara, CA, USA) in positive ion mode, scanning from *m*/*z* 100 to 1100. MS parameters included a drying gas flow of 8.0 L/min, gas temperature of 320 °C, nebulizer pressure of 35 psi, sheath gas temperature of 350 °C, sheath gas flow of 11 L/min, capillary voltage of 3.5 kV, skimmer voltage of 65 V, OCT RFV of 750 V, and fragmentor voltage of 175 V. A quality control (QC) sample, made by pooling 5 μL from each of the 49 samples, was analyzed after every 10 samples to monitor system stability and data reliability.

### 2.4 GC-MS-based untargeted metabolomics analysis

#### 2.4.1 Sample preparation

The volatile oils were extracted via hydrodistillation, following a modified procedure reported by Zhao et al. [17]. All *L.* *aggregata* samples were ground into a fine powder and passed through a 24-mesh sieve for use in the experiment. A 25.0 g of *L. aggregata* powder was introduced into a steam distillation apparatus and ultrapure water, and the powder was allowed to soak for 3 h. Subsequently, the mixture was distilled using the steam distillation method for 6–8 h until no further increase in volatile oils was observed. The volatile oils were then collected, and any residual water was evaporated. The concentrated oils were transferred to a centrifuge tube and stored at cold temperatures for subsequent use.

#### 2.4.2 GC-MS analysis

The volatile oil was characterized using an Agilent 5977B GC/MSD system (Agilent Technologies, Santa Clara, CA, USA), which was fitted with a 30 m × 0.32 mm × 1 μm HP-5MS (5% phenylpolymethylsiloxane) capillary column. The oven temperature profile commenced at 180 °C for 10 min, followed by a linear ramp to 250 °C over 2 min at a rate of 20 °C/min. The injection volume was set to 2.0 μL with a split ratio of 1:5. The injector temperature was maintained at 250 °C. Helium, with a purity of ≥99.999 %, served as the carrier gas at a constant flow rate of 2.0 mL/min. Mass spectra were recorded in electron impact mode at an electron energy of 70 eV. The ion source and MS quadrupole temperatures were adjusted to 230 and 150 °C, respectively. Data acquisition was performed in full-scan mode over a mass range of *m/z* 30.0–800.0. QC sample prepared by mixing 5 μL of volatile oil from 49 samples was injected at intervals of every 10 samples throughout the analysis (5 times).

### 2.5 Metabolomics analysis

We used Profinder software to process raw UPLC-QTOF-MS data, including peak extraction, alignment, and identification, after normalizing peak intensities with batch recursive feature extraction. The Unknowns Analysis software extracted features from GC-MS data using the SureMass algorithm and identified them with the NIST 17 database. The data were then converted to CEF format for analysis in Mass Profiler Professional Version 15.0, where multivariate techniques, such as principal component analysis (PCA), partial least squares discriminant analysis (PLS-DA), and hierarchical cluster analysis (HCA), were applied to differentiate between *L. aggregata* root types. The analysis parameters included a minimum response value of 5000 counts, a minimum of 2 ions per compound, exclusion of multi-charge compounds, and normalization. The compound occurrence frequency was set at 80%. Analysis of variance (ANOVA) (*P* < 0.05) and a fold change value (FC > 2.0) were used to identify significant differences among sample groups.

### 2.6 Hyperspectral imaging system and image acquisition

Three samples were randomly selected from each batch of *L. aggregata* samples (49 batches) and were respectively powdered and sieved by a 60-mesh sieve. A total of 147 samples were used for hyperspectral image acquisition, including tuberous roots (*n* = 87), taproots (*n* = 30), and old roots (*n* = 30). We weighed 2 g of each sample powder and put it in a plastic petri dish, and placed it on a black background plate. The sample's surface should be flat and free from unevenness to avoid light shadow, and the thickness of the sample should be uniform (usually 1–5 mm). Then, it was scanned with a FigSpec Hyperspectral Camera FS-13 from Hangzhou Color Spectrum Technology Co., Ltd. (Hangzhou, China). The camera's detection wavelengths ranged from 400 to 1000 nm, with settings including an exposure time of 11,000 μs, a focal length of 25.00 mm, a scanning distance of 0.45 m, and a speed of 30 mm/s. The light source and measurement were synchronized with white calibration enabled at a reflectivity coefficient of 80.00%. Illumination was adjusted for clear images.

HSI data, which includes signals from samples, the environment, and instruments, underwent reflectance calibration to remove non-sample influences. Calibration involved black-and-white corrections: the white balance image (W) was based on reflectance values from a Teflon white surface, and the dark image (D) was captured with the light off and the lens covered. The calibration image (I) was calculated using the following Eq. (1):

Eq. 1 $$\begin{array}{c} I=\frac{I_{0}-D}{W-D}\times 100 \end{array}$$

Where I represents the corrected reflectance hyperspectral image in a unit of relative reflectance (%); $I_{0}$ represents the raw hyperspectral image; D stands for the dark image (0% reflectance); and W is the white reference image (100% reflectance) [18]. Hyperspectral data were extracted from calibrated images using FigSpec software to define the region of interest (ROI), taking into account surface unevenness. The average spectrum from the ROI provided raw spectral data for *L. aggregata* root samples.

### 2.7 Determination of multi-indicators of Linderae Radix

The Agilent 1260 series UPLC system (Agilent Technologies, Santa Clara, CA, USA) quantified the concentrations of norisoboldine, linderane, and lindenenol on an Eclipse XDB-C18 column (4.6 mm × 250 mm, 5 μm; Agilent Technologies, Santa Clara, CA, USA). The mobile phases consisted of water with 0.5% formic acid, 0.1% triethylamine (A) and acetonitrile with 0.5% formic acid (B). The gradient elution program was: 0–20 min, 10% B; 20–21 min, 10%–65% B; 21–35 min, 65%–75% B; 35–37 min, 10% B. The flow rate was 0.80 mL/min, the column temperature was 30 °C, the injection volume was 10 μL, and the ultraviolet (UV) detection was 235 nm. Calibration was performed using standard solutions, and the peak areas were plotted against mass concentrations to construct standard curves. Detailed results are in Table S2. To ensure the repeatability, accuracy, and applicability of the quantitative analysis method, precision tests, stability tests, repeatability tests, and sample recovery tests were conducted on the instrument throughout the day, as detailed in Tables S3–S7. Sample preparation involved filtering supernatants through a 0.45 μm membrane, and the content of each constituent was calculated using the respective standard curve.

### 2.8 HSI data analysis

#### 2.8.1 Spectral data pre-processing and effective wavelengths (EWs) selection

To enhance spectral quality and mitigate interference effects, multiple preprocessing techniques were applied. Standard normal variate (SNV) was employed to remove the effect of scattering from the sample surface and reduce the intensity differences in the spectrum. Savitzky-Golay (SG) smoothing is often applied to reduce high-frequency noise generated by instruments. First-order derivative (1^st^D) is a standard technique for eliminating the additive scattering, while the second derivative (2^nd^D) resolved multiplicative scattering [19].

Feature wavelength selection is employed to filter out the EWs from the entire spectrum to streamline the modeling process and enhance detection efficiency. In this study, three methods—competitive adaptive reweighted sampling (CARS), successive projections algorithm (SPA), and random frog (RF)—were utilized to identify critical wavelengths that have a strong correlation with the measurement indicators. For wavelength selection, the choice of algorithms was driven by their strengths: CARS provides high stability and reliability, making it ideal for identifying EWs tied to active components [20]. RF is favored for its global optimization and ability to capture interactive effects, making it effective for distinguishing spectral differences across groups [21]. SPA excels in addressing multicollinearity and efficiently extracting the most representative bands [22].

#### 2.8.2 Modeling analysis

Based on the pre-processing of NIRS information, a multi-layer perceptron (MLP), logistic regression (LR), and support vector machine (SVM) were applied in this study to classify different root types of *L. aggregata*. MLP is highly effective at learning complex patterns and adapting to intricate feature relationships [23]. LR, on the other hand, is simple, efficient, and interpretable, offering a probabilistic approach [24]. SVM is well-suited for handling high-dimensional data efficiently, due to its memory efficiency and versatility in both classification and regression tasks [25].

Regarding the quantitative models, extreme learning machine (ELM) has shown the advantages of fast learning speed and good generalization ability [26]. Least squares support vector machine (LSSVM) is optimal for solving linear and nonlinear problems quickly and effectively [27]. Backpropagation neural network (BPNN) is easy to program and apply, making it suitable for complex modeling tasks [28].

For classification, the dataset (*n* = 147) was partitioned into training (60%, *n* = 87), validation (20%, *n* = 30), and test sets (20%, *n* = 30) using stratified randomization. 5-fold cross-validation applied. CARS was used for variable selection (MCCV: 1000 runs, 80% calibration, center; max LV = 14; 50 Monte Carlo samplings with 10-fold CV), retaining variables with the lowest RMSECV based on PLS coefficient weighting and exponential reduction. SPA was applied for efficient spectral variable selection (dataset split by SPXY, test ratio = 1/3; standardization applied; max candidate variables m_max = 50; final subset determined by minimizing PRESS and F-test at α = 0.25). The Random Frog algorithm was run for 2000 iterations (*n* = 2000), with an initial model containing 2 variables (Q = 2). For quantitative modeling, data were split 2:1. 10-fold cross-validation applied. LSSVM used RBF kernel (γ = σ^2^ = 2). BPNN had a single hidden layer and 8 hidden neurons (1000 epochs, η = 0.6, target MSE = 1 × 10^−5^). ELM employed 40 sigmoid nodes. MLP had a single hidden layer with 50 neurons.

#### 2.8.3 Model evaluation

The coefficients of determination for calibration ($R_{c}^{2}$) evaluated the performance of these models, root mean square error of calibration (RMSEC), coefficients of determination for prediction ($R_{p}^{2}$), root mean square error of prediction (RMSEP), and the ratio of prediction to deviation (RPD). RPD is a crucial indicator for evaluating model performance. Typically, RPD values between 1.5 and 2 indicate predictive solid ability, while values below 1.5 indicate poor predictive quality. Robust models have high correlation coefficients ($R_{c}^{2}$ and $R_{p}^{2}$) high RPDs, and low errors (RMSEC and RMSEP). The performance metrics were calculated using the following Eqs. Eq. 2, Eq. 3, Eq. 4):

Eq. 2 $$\begin{array}{c} R^{2}=1-\frac{\sum_{i=1}^{n}{\left(y_{i}-\hat{y_{i}}\right)}^{2}}{{\sum_{i=1}^{n}\left(y_{i}-\bar{y}\right)}^{2}} \end{array}$$

Eq. 3 $$\begin{array}{c} \mathrm{RMSE}=\sqrt{\frac{\sum_{i=1}^{n}{\left(\hat{y_{i}}-y_{i}\right)}^{2}}{n}} \end{array}$$

Eq. 4 $$\begin{array}{c} \mathrm{RPD}=\frac{\sigma_{y}}{RMSEP} \end{array}$$

where, $y_{i}$, $\hat{y_{i}}$, $\bar{y}$, and $\sigma_{y}$ represent the measured, predicted, mean, and standard deviation values of the response variables, respectively [14].

For root type discrimination, model performance was typically evaluated using standard metrics, such as accuracy, precision, recall, F1-score and kappa. The definitions of these metrics are provided in Eqs. Eq. 5, Eq. 6, Eq. 7, Eq. 8, Eq. 9):

Eq. 5 $$\begin{array}{c} \mathrm{Accuracy}=\frac{TP+TN}{TP+FP+TN+FN} \end{array}$$

Eq. 6 $$\begin{array}{c} \mathrm{Precision}=\frac{TP}{TP+FP} \end{array}$$

Eq. 7 $$\begin{array}{c} \mathrm{Recall}=\frac{TP}{TP+FN} \end{array}$$

Eq. 8 $$\begin{array}{c} F1-score=\frac{2\times Precision\times Recall}{Precision+Recall} \end{array}$$

Eq. 9 $$\begin{array}{c} K=\frac{P_{o}-P_{e}}{1-P_{e}} \end{array}$$

where TP, TN, FP, and FN represent true positives, true negatives, false positives, and false negatives, respectively. $P_{o}$ represented the proportion of consistency between the predicted and actual labels of the model across all categories. $P_{e}$ represented the sum of the product of the proportion predicted by the model for that category and the true proportion of that category. $1-P_{e}$ represented the difference between the maximum possible consistency and the expected consistency [29].

#### 2.8.4 Visualization of the norisoboldine distribution and merge in slices

The prediction outcomes were then visualized through pseudo-color mapping, effectively displaying the distribution patterns of various indicator levels. Using ZEISS Efficient Navigation (ZEN) software, the parameters of the shot pictures are adjusted, and then the images are merged.

#### 2.8.5 Software

In this study, we utilized the software FigSpec, which accompanies the HSI system, to perform black and white corrections, extract ROIs, and acquire spectral reflectance from the powder and slice samples of *L. aggregata* hyperspectral images. The classification model is constructed using the PyCharm Community Edition 2024.2.1 software. The Unscrambler 10.4 was used for pre-processing the spectral data, while MATLAB R2018b was employed to screen the spectral data for characteristic bands and regression modeling. Imaging visualization and merge use Python 3.11 and ZEN software, respectively.

## 3 Results and discussion

### 3.1 UPLC-QTOF-MS-based metabolites profiling of *L. aggregata*

#### 3.1.1 Overall metabolic profile analysis and identification of compounds in *L. aggregata* methanol extract

The total ion current (TIC) plots for all QC samples acquired in electrospray ionization positive mode (ESI^+^) are presented in Fig. S1. By overlaying the TIC plots separately, including QC, and comparing retention time and peak intensity, it was observed that the TIC plots of each sample group are highly similar, with only minor variations in these two parameters. This similarity suggests that the instrument-induced error in our experiments was minimal.

A total of 1587 components were detected in ESI^+^ mode across all *L. aggregata* samples, with 66 metabolites identified using our self-constructed library of *L. aggregata* chemical components combining reference materials with literature-based data, which are detailed in Table S8. These metabolites were categorized into three major groups based on chemical classification. Sesquiterpenes, numbering 35, constituted 53.03% of the identified metabolites, followed by alkaloids (20 metabolites, 30.30%) and unclassified metabolites (8 metabolites, 12.12%). Two flavonoids and one tannin were also identified.

#### 3.1.2 Multivariate data analyses of the UHPLC-QTOF-MS dataset

##### 3.1.2.1 PCA

**Fig. 2:**
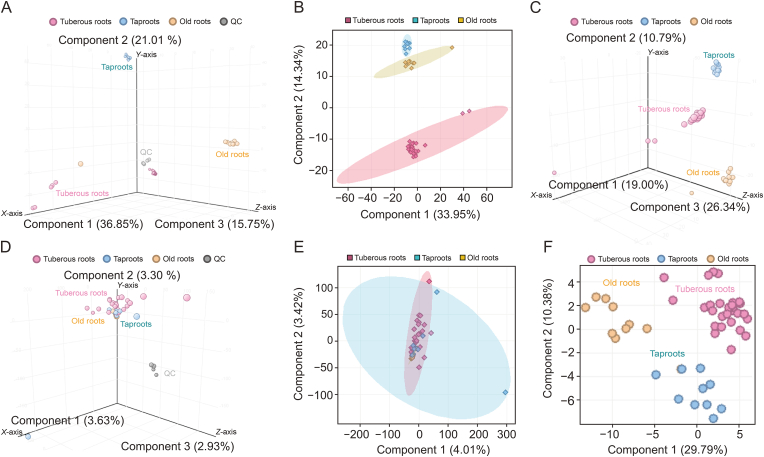
Multivariate statistical analysis showing the specificity of three root types of *L. aggregata* samples (*n* = 49) by ultra-performance liquid chromatography-quadrupole time-of-flight mass spectrometry (UPLC-QTOF-MS) and gas chromatography-mass spectrometry (GC-MS). (A) Principal component analysis (PCA) 3D scores plot the complete data set with quality control (QC) of UPLC-QTOF-MS. (B) PCA scores plot of the data set, excluding QC of UPLC-QTOF-MS. (C) Score plots of partial least squares discrimination analysis (PLS-DA) of UPLC-QTOF-MS. (D) PCA 3D scores plot the complete data set with QC of GC-MS. (E) PCA scores plot of the data set, excluding QC of GC-MS. (F) Score plots of PLS-DA of GC-MS. QC: quality control.

PCA is typically the initial method employed to evaluate the extent of variance among different sample groups and their overall distribution [30]. It was conducted to provide an overview of the metabolomics data, with the results of the three dimensional (3D) PCA plot shown in Fig. 2A. The 6 QC samples, depicted in grey, are closely clustered in the central region, indicating good experimental reproducibility. The PCA was conducted on all *L. aggregata* samples, and the resulting scores are plotted in Fig. 2B. This figure demonstrates that principal components (PC) 1 and PC2 account for 48.29% of the total variance, with PC1 explaining 33.95% and PC2 explaining 14.34%. Analysis of Fig. 2B reveals that the sample groups from the three distinct root types of *L. aggregata* (tuberous root, taproot, and old root) are clustered in separate regions, suggesting significant differences. Additionally, the old root and taproot groups are clustered in a smaller area, indicating that the differences between these two groups are relatively minor.

##### 3.1.2.2 PLS-DA

PLS-DA was conducted to identify differential metabolites associated with different sample groups within the dataset. The scores from the PLS-DA model for all sample groups are depicted in Fig. 2C, which clearly shows the model's ability to distinguish between various *L. aggregata* samples, with the distribution of differences among sample groups being evident.

R^2^X and R^2^Y represent the percentage of X and Y matrix information explained by the PLS-DA classification model, while Q^2^Y, calculated through cross-validation, assesses the model's predictive power. The model evaluation parameters, R^2^ (R^2^X and R^2^Y) and Q^2^ are presented in Table S9 and Fig. S2. The closer these values are to 1, the more stable the model. Generally, a model is considered highly stable if Q^2^ > 0.5, more stable if Q^2^ is between 0.3 and 0.5, and less reliable if Q^2^ < 0.3. An increase in model complexity, accompanied by additional components, can lead to an increase or stabilization of R^2^ and a potential decrease in Q^2^, indicating overfitting [31]. Table S9 and Fig. S2 demonstrate that our model is not overfitted, exhibiting high stability with three components, with R^2^X, R^2^Y, and Q^2^ values of 0.561, 0.993, and 0.848, respectively.

##### 3.1.2.3 HCA of differential metabolites

In metabolomics, metabolites are considered differential if they have an FC value > 2 and a *P* value < 0.05. We identified 230 differential metabolites under ESI^+^, with 25 confirmed from our *L. aggregata* library, as detailed in Table 1. HCA and a heatmap (Fig. S3) visualized these metabolites, with color bars indicating FC values from minimum (blue) to maximum (red), showing high similarity within clusters.

**Table 1:** The identified differential metabolites for distinguishing different roots’ types of *L. aggregata* by ultra-performance liquid chromatography-quadrupole time-of-flight mass spectrometry (UPLC-QTOF-MS) and gas chromatography-mass spectrometry (GC-MS).

No.	Retention time (min)	Compound	Chemical formula	CAS
Non-volatile metabolites				
1	0.9964	Linderanolide G	C_16_H_22_O_5_	1346220-70-9
2	1.0340	Linderolide C or its isomer	C_15_H_18_O_4_	1346458-51-2
3	1.0660	Dehydrolindestrenolide	C_15_H_16_O_2_	32810-35-8
4	1.6543	Northalifoline	C_10_H_11_NO_3_	157669-72-2
5	3.8464	Linderaline or its isomer	C_18_H_19_NO_4_	731851-55-1
6	4.2950	*N*-*trans*-feruloyltyramine or its isomer	C_18_H_19_NO_4_	66648-43-9
7	4.6040	(+)-Coclaurine	C_17_H_19_NO_3_	2196-60-3
8	5.6520	Boldine or its isomer	C_19_H_21_NO_4_	476-70-0
9	6.0500	Aggreganoid F	C_31_H_36_O_5_	2375675-51-5
10	7.2040	*N*-*trans*-feruloylmethoxytyramine	C_19_H_21_NO_5_	78510-19-7
11	7.7810	Linderanoid M isomer	C_32_H_36_O_6_	–
12	7.8090	(+)-Norboldine or its isomer	C_18_H_19_NO_4_	5890-18-6
13	9.1575	(+)-Norboldine acetate	C_20_H_21_NO_5_	1130234-57-9
14	9.3551	Linderanoid O	C_34_H_38_O_8_	2831405-75-3
15	10.7080	Salutaridine or its isomer	C_19_H_21_NO_4_	1936-18-1
16	10.9250	Hernangerine or its isomer	C_18_H_17_NO_4_	31520-97-5
17	11.2840	Epicatechin-(4β-8,2-O-7)-epicatechin	C_30_H_24_O_12_	–
18	12.3560	Aggreganoid C	C_33_H_40_O_4_	2369632-30-2
19	12.7664	Linderagalactone C or its isomer	C_15_H_18_O_4_	1174550-75-4
20	13.2333	Aggreganoid E isomer	C_31_H_34_O_5_	–
21	13.3740	(−)-8β-(4′-hydroxybenzyl)-2-methoxyberbin-3,10,11-triol isomer	C_25_H_25_NO_5_	–
22	15.7746	Linderagalactone A	C_15_H_19_ClO_4_	1174550-73-2
23	17.7099	Linderolide E or its isomer	C_15_H_18_O_3_	1346458-54-5
24	18.4767	Lindenenol (linderene)	C_15_H_18_O_2_	26146-27-0
25	18.6124	Shizukanolide or its isomer	C_15_H_18_O_2_	70578-36-8
Volatile metabolites				
1	2.4896	2-Butanone	C_4_H_8_O	78-93-3
2	2.8574	D-Limonene	C_10_H_16_	5989-27-5
3	3.1273	Benzaldehyde, 4-methyl-	C_8_H_8_O	104-87-0
4	3.5096	3-Methylbenzyl alcohol	C_8_H_10_O	587-03-1
5	3.8333	(−)-Borneol	C_10_H_18_O	464-45-9
6	3.9485	L-α-Terpineol	C_10_H_18_O	10482-56-1
7	4.0352	Bicyclo[3.1.0]hexan-3-ol, 4-methylene-1-(1-methylethyl)-, acetate	C_12_H_18_O_2_	3536-54-7
8	4.6697	(−)-Bornyl acetate	C_12_H_20_O_2_	5655-61-8
9	5.0731	2-Oxabicyclo[2.2.2]octan-6-ol, 1,3,3-trimethyl-, acetate	C_12_H_20_O_3_	72257-53-5
10	5.3877	Nonanoic acid	C_9_H_18_O_2_	112-05-0
11	5.6837	6,7-Dimethyl-1,2,3,5,8,8a-hexahydronaphthalene	C_12_H_18_	–
12	5.7314	δ-EIemene	C_15_H_24_	20307-84-0
13	5.7434	(−)-β-Elemene	C_15_H_24_	515-13-9
14	5.8754	2,5-Dimethoxy-*p*-cymene	C_12_H_18_O_2_	14753-08-3
15	6.2169	Kumarone	C_11_H_14_O	34545-88-5
16	6.4858	α-Guaiene	C_15_H_24_	3691-12-1
17	6.6316	Undecanoic acid	C_11_H_22_O_2_	112-37-8
18	6.7323	1,4,7,-Cycloundecatriene, 1,5,9,9-tetramethyl-, Z,Z,Z-	C_15_H_24_	400822-79-9
19	6.7458	Cadina-3,5-diene	C_15_H_24_	267665-20-3
20	6.8184	3,4-Dehydro-β-ionone	C_13_H_18_O	1203-08-3
21	6.9099	γ-Muurolene	C_15_H_24_	30021-74-0
22	7.1153	1,5-Cadinadiene	C_15_H_24_	483-75-0
23	7.2176	(±)-γ-Cadinene	C_15_H_24_	39029-41-9
24	7.2800	Curcumene	C_15_H_22_	644-30-4
25	7.3108	2-Isopropenyl-4a,8-dimethyl-1,2,3,4,4a,5,6,8a-octahydronaphthalene	C_15_H_24_	207297-57-2
26	7.6064	(+)-δ-Cadinene	C_15_H_24_	483-76-1
27	7.8071	*l*-Calamenene	C_15_H_22_	483-77-2
28	7.8146	Phenol, 4,6-di(1,1-dimethylethyl)-2-methyl-	C_15_H_24_O	616-55-7
29	8.2609	Curzerene	C_15_H_20_O	17910-09-7
30	8.3143	β-Calacorene	C_15_H_20_	50277-34-4
31	8.3564	Selina-3,7(11)-diene	C_15_H_24_	6813-21-4
32	10.2584	Ethylbenzene	C_8_H_10_	100-41-4
33	12.8047	Metacrate	C_9_H_11_NO_2_	1129-41-5
34	14.6557	Succinic acid, 2-chloro-6-fluorophenyl 4-methoxybenzyl ester	C_18_H_16_ClFO_5_	–
35	15.4825	1,4-Cyclohexadiene, 3,3,6,6-tetramethyl-	C_10_H_16_	2223-54-3
36	15.9466	Succinic acid, 3-chlorophenyl 4-methoxybenzyl ester	C_18_H_17_ClO_5_	–
37	16.1884	Benzenemethanol, 4-methyl-α-(1-methyl-2-propenyl)-, (*R*∗,*R*∗)-	C_12_H_16_O	83173-76-6
38	16.6639	2,3,4-Trifluorobenzoic acid, 2-chloroethyl ester	C_9_H_6_ClF_3_O_2_	–
39	17.3030	1,2,3,4,5-Pentamethoxybenzene	C_11_H_16_O_5_	13909-75-6
40	17.7138	Diisobutyl phthalate	C_16_H_22_O_4_	84-69-5
41	20.5730	Seselin	C_14_H_12_O_3_	523-59-1
42	21.3095	p-Aminotoluene	C_7_H_9_N	106-49-0
43	21.3308	Shizukanolide	C_15_H_18_O_2_	70578-36-8
44	21.3729	Gazaniolide	C_15_H_18_O_2_	71609-02-4
45	21.6300	3-(4-Methoxyphenoxy)benzoic acid	C_14_H_12_O_4_	117423-75-3
46	23.2167	2-(4a,8-Dimethyl-2,3,4,4a,5,6-hexahydro-naphthalen-2-yl)-prop-2-en-1-ol	C_15_H_22_O	–
47	23.2394	Tetracyclo[6.2.1.1(3,6).0(2,7)]dodec-4-ene, 11-isopropylidene-	C_15_H_20_	–
48	23.6626	6-Octen-1-one, 3-ethenyl-1-phenyl-	C_16_H_20_O	65564-67-2

−: no data.

The analysis confirmed discrete clusters for *L. aggregata*'s tuberous root, taproot, and old root samples, with taproot and old roots exhibiting more significant variation in component content than tuberous roots. The heatmap highlighted 25 significantly differential metabolites, with sesquiterpenes and alkaloids showing substantial response value differences among the three root types. Key compounds characterizing the taproot and old root groups included (+)-coclaurine, hernangerine, epicatechin-(4β-8,2-O-7)-epicatechin, aggreganoid C, (+)-norboldine acetate, dehydrolindestrenolide, shizukanolide, linderanolide G, linderoline E, and their isomers. In contrast, the old root group was defined by boldine, *N*-*trans*-feruloyl tyramine, linderanoid M, linderolide C, and their isomers. The tuberous root group featured a high content of linderaline or its isomer, lindenenol, linderanoid O, and *N*-*trans*-feruloylmethoxythramine, among others.

### 3.2 GC-MS-based volatile oil metabolites profiling of *L. aggregata*

#### 3.2.1 Overall metabolic profile analysis and identification of compounds in *L. aggregata* volatile oil

GC-MS raw data processing revealed that 49 batches of volatile oil samples were matched with 56,299 volatile components, with 17,891 components characterized (Table S10). The TIC profiles for the volatile oil samples are depicted in Fig. S4, demonstrating that the ion flow diagrams across the three sample groups are mainly consistent with minor variations. The smooth chromatographic peak baselines suggest excellent instrument stability, enhancing the instrumental analyses’ reliability and data outcomes.

#### 3.2.2 Multivariate data analyses of the GC-MS dataset

##### 3.2.2.1 PCA

Fig. 2D presents the PCA 3D plots for the three root sample groups, with the 5 QC samples (in grey) tightly clustered in the central region, indicating the high reproducibility of the conducted experiments. The PCA model scores depicted in Fig. 2E indicate that the three groups of samples do not differ significantly, with some overlap. This is likely due to the similarity in the types of volatile components across different parts of *L. aggregata* and the lack of significant differences in the relative contents of most volatile components. Zhao et al. [17] have previously demonstrated minimal variation in the content and composition of the main components of volatile oils between the taproots and tuberous roots of *L. aggregata*. Since PCA reflects the overall sample composition, the lack of clear distinction in the PCA scores is consistent with these findings.

##### 3.2.2.2 PLS-DA

Fig. 2F illustrates the PLS-DA model scores, positioning the old root group on the left, the tuberous root group on the right, and the taproot group on the lower side, accentuating the differentiation effect. Given the modest differences in volatile oil composition among the three groups, which would not benefit from additional components and risk overfitting, only two were retained. This PLS-DA analysis captured two principal components, explaining 29.79 % and 10.38 % of the variance. With R^2^X = 0.402, R^2^Y = 0.795, and Q^2^ = 0.577, the Q^2^ value exceeding 0.5 signifies a robust predictive performance of the PLS-DA model (Table S9 and Fig. S5).

##### 3.2.2.3 HCA of differential metabolites

A total of 100 differential metabolites were identified using criteria of an FC value greater than 2 and a *P* value less than 0.05, of which 48 were confirmed by matching against the NIST17 database (Table 1). A comprehensive analysis of these differential metabolites (Fig. S6A) revealed that the contents of volatile components in different root samples are different. In general, most volatile components in the old roots of *L. aggregata* had significantly lower response values compared to those in tuberous roots and taproots. This could be because both the tuberous and taproots are in an active growth phase, where secondary metabolism is more vigorous. At the same time, the metabolic activity in old roots declines with age, leading to reduced synthesis of volatile oils. Moreover, all components in tuberous roots showed a moderate response compared to the other two root types, neither too low nor too high. In detail, further analysis of the 48 identifiable volatile constituents (Fig. S6B) showed that, except 2,3,4-trifluorobenzoic acid, 2-chloroethyl ester, curcumene, seselin, nonanoic acid, diisobutyl phthalate and undecanoic acid, the relative content of all other differential metabolites in the old root group was significantly lower than that in the tuberous root group. Additionally, metabolites such as gazaniolide, 1,5-cadinadiene, kumarone, δ-elemene, succinic acid, 2-chloro-6-fluorophenyl 4-methoxybenzyl ester, bicyclo[3.1.0]hexan-3-ol, 4-methylene-1-(1-methylethyl)-, acetate, selina-3,7(11)-diene, 3,4-dehydro-β-ionone, curzerene, 2-oxabicyclo[2.2.2]octan-6-ol, 1,3,3-trimethyl acetate, and 1,4,7-cycloundecatriene, 1,5,9,9-tetramethyl (Z,Z,Z-) in the taproot group had lower response values than those in the tuberous root group.

### 3.3 Spectral analysis

#### 3.3.1 Spectral characteristics of *L. aggregata* roots and pre-processing

**Fig. 3:**
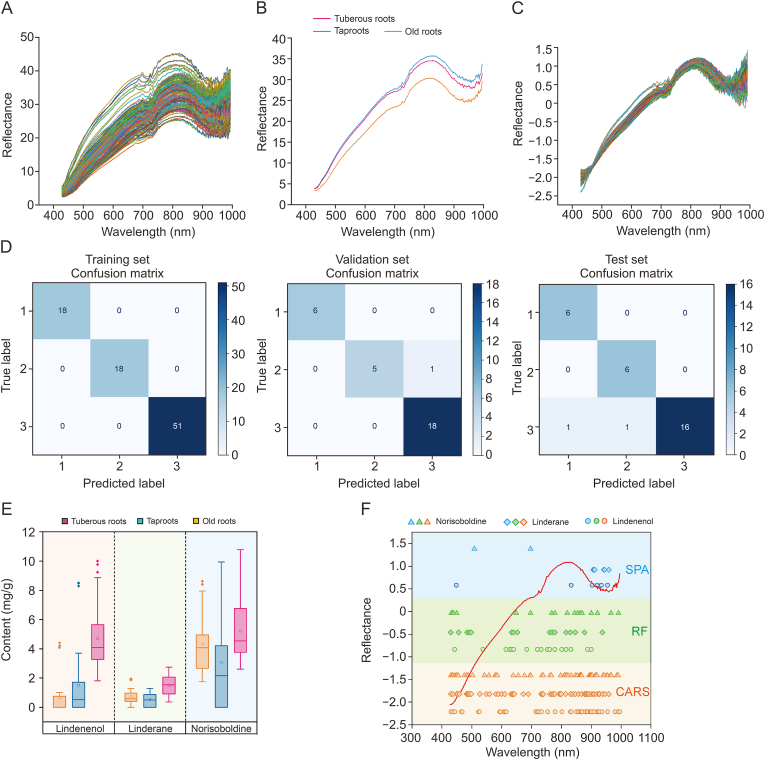
The quantitative data and spectra information analysis of *L. aggregata*. (A) Spectral curves for all samples. (B) Average raw spectral reflectance of the *L. aggregata* samples from tuberous roots, taproots, and old roots. (C) Visible (VIS)-near-infrared (NIR) optimized by standard normal variate (SNV). (D) The confusion matrix of training/validation/test set for the classification results (where labels 1, 2, and 3 represent taproot, old root, and tuberous root, respectively). (E) Box-plot analyses the three main constituents' contents in *L. aggregata*. (F) Specific locations of important wavelengths were extracted based on the VIS-NIR data. CARS: competitive adaptive reweighted sampling; RF: random frog; SPA: successive projections algorithm.

In this research, we focused on the 270 spectral bands ranging from 427 to 994 nm for subsequent analysis, as the spectral curve exhibited considerable noise at its extremities. Fig. 3A presents the complete spectral curves of all samples, with the mean spectral data for various types of *L. aggregata* root samples detailed in Fig. 3B, namely tuberous roots, taproots, and old roots. The average spectra of these three sample categories, as depicted in Fig. 3B, reveal similar patterns, with reflectance values ascending as the wavelength increases from 427 nm to 994 nm. Notably, *L. aggregata*'s old roots' spectral reflectance was markedly lower than that of tuberous roots and taproots. However, the characteristic peaks, indicative of individual bands, were consistent across the spectral curves of the three root types of *L. aggregata*. These curves show an initial increase to a plateau followed by a decrease, with more pronounced fluctuations observed in the NIR region (720–994 nm) than in the VIS region (427–720 nm). These raw spectra indicate a significant rise in reflectance values between 720 and 994 nm, which corresponds to the third overtone region associated with the absorptions of hydrogen-bonded O–H and C–H groups. The spectral variations within the VIS range (427–720 nm) are primarily ascribed to the color characteristics of the samples [14].

Appropriate spectral pre-processing techniques are crucial for mitigating the influence of non-target factors on detected signal information and enhancing valuable spectral absorption features [32]. In this study, we employed methods such as SNV, 1^st^D, 2^nd^D, and SG smoothing, individually or in combination, to optimize a stable and reliable model and extend its applicability. Fig. 3C presents the pre-processed spectra for all samples. Compared to the original raw spectra, these pre-treated spectra showed an improved signal-to-noise ratio and significantly reduced noise interference. The subsequent model results indicated that the SNV pre-processing method provided the most exceptional predictive performance.

#### 3.3.2 Establishment of classification model using different machine learning methods

While spectral variations exist among the three root types of *L. aggregata*, visual inspection cannot distinguish them, as depicted in Fig. S7. Therefore, selecting the appropriate classification technique for precise identification is crucial. In our HSI analysis of *L. aggregata* root types, we employed SVM, LR, and MLP models with various spectral pre-processing techniques, such as SG smoothing, SNV, 1^st^D, 2^nd^D, 1^st^D-SG, and 2^nd^D-SG (results detailed in Table 2). Combinations of SNV with SG smoothing, 1^st^D, or 2^nd^D were evaluated to optimize signal-to-noise ratios. These methods were systematically compared to identify the optimal preprocessing strategy for minimizing spectral artifacts and improving model robustness. The best predictive performance within the VIS-NIR spectrum was obtained using the LR model with 2^nd^D-SG processed spectra, achieving a 100%, 96.67% and 93.33% accuracy rate in training, validation and test sets. And Fig. 3D shows the confusion matrix of model training, validation, and test results, which is used to evaluate the classification performance of the model, and shows the relationship between the test results of all categories and the actual results in matrix form. The superior performance of LR in this study can be attributed to its simplicity, strong interpretability, high training efficiency, and the fact that it produces a probabilistic output, which is particularly advantageous for small-scale datasets [24]. LR performs exceptionally well when the sample size is relatively small, as in this experiment with 147 batches and only three characteristic components, as it is less prone to overfitting and can be trained more efficiently. In contrast, SVM and MLP have distinct advantages: SVM's ability to handle high-dimensional data and MLP's flexibility in fitting complex, non-linear relationships [25,33]. However, both models require a larger sample size to fully exploit their strengths. In the current study, the limited sample size and low feature dimension resulted in these models struggling to outperform the simpler LR model. As the sample size increases, we anticipate that SVM and MLP may offer improved performance, particularly in handling more complex data structures.

**Table 2:** Accuracy of classification of *L. aggregata* roots (data were split into training/validation/test sets of ratio 60/20/20).

Models	Types of data set and cross-validation	Pre-processing methods						
		Raw	SG	SNV	1^st^D	2^nd^D	1^st^D-SG	2^nd^D-SG
SVM	Training set (%)	100.00	100.00	100.00	100.00	100.00	100.00	**100.00**
	Validation set (%)	70.00	66.67	70.00	83.33	83.33	83.33	**83.33**
	Test set (%)	60.00	66.67	60.00	76.67	80.00	83.33	**83.33**
	Cross-validation (%)	80.36	58.22	79.46	80.40	77.79	82.86	**78.66**
LR	Training set (%)	100.00	100.00	100.00	100.00	100.00	100.00	**100.00**
	Validation set (%)	93.33	86.67	96.67	96.67	93.33	96.67	**96.67**
	Test set (%)	86.67	90.00	90.00	83.33	80.00	93.33	**93.33**
	Cross-validation (%)	94.06	88.91	94.06	91.52	87.25	93.15	**86.30**
MLP	Training set (%)	80.46	41.38	78.16	56.32	**93.10**	77.01	50.57
	Validation set (%)	62.07	24.14	55.17	37.93	**72.41**	44.83	27.59
	Test set (%)	53.33	40.00	63.33	40.00	**70.00**	50.00	26.67
	Cross-validation (%)	64.34	56.16	61.70	68.57	**76.00**	68.48	78.00

SG: Savitzky-Golay; SNV: standard normal variate; 1^st^D: first-order derivativ; 2^nd^D: second derivative; SVM: support vector machine; LR: logistic regression; MLP: multi-layer perceptron.

Table 3 shows the performance indicators and model complexity test results of 2^nd^D-SG-SVM, 2^nd^D-SG-LR, and 2^nd^D-MLP models. By measuring the recognition performance from different angles, the 2^nd^D-SG-LR model outperforms other models in all indicators. The precision, recall, F1-score, and Kappa in the test set are 94.29%, 93.33%, 93.39%, and 88.64%, respectively. From the above analysis, it can be seen that the 2^nd^D-SG-LR model performs well in all indexes, and has higher recognition performance and generalization ability. Collectively, our machine-learning-augmented HSI successfully differentiated the three *L. aggregata* root types, with the chemical distinctions further validated by the non-targeted metabolomics analysis conducted via LC-MS and GC-MS.

**Table 3:** The performance of optimal classification models in differentiating among *L. aggregata* with different root types.

Models	Types of data set	Performance			
		Precision	Recall	F1-score	Kappa
2^nd^D-SG-SVM	Training set (%)	100.00	100.00	100.00	100.00
	Validation set (%)	86.96	83.33	82.02	66.22
	Test set (%)	86.96	83.33	82.02	66.22
**2^nd^D-SG-LR**	Training set (%)	**100.00**	**100.00**	**100.00**	**100.00**
	Validation set (%)	**96.84**	**96.67**	**96.56**	**93.90**
	Test set (%)	**94.29**	**93.33**	**93.39**	**88.64**
2^nd^D-MLP	Training set (%)	93.60	93.10	93.15	91.97
	Validation set (%)	74.05	72.41	72.85	63.75
	Test set (%)	69.52	70.00	68.38	67.51

SG: Savitzky-Golay; 2^nd^D: second derivative; SVM: support vector machine; LR: logistic regression; MLP: multi-layer perceptron.

#### 3.3.3 Quantification analysis of the three indicators

##### 3.3.3.1 Selection of the three main quantitative components

To assess the quality of *L. aggregata in situ*, a further investigation was conducted to examine the relationship between chemical constituents and hyperspectral images. Three components, including norisoboldine, linderane, and lindenenol, were selected for quantification as typical volatile and non-volatile compounds. These three components are also chosen because noisoboldine and linderane used as index components of *L. aggregata* in the Chinese Pharmacopoeia (2020) [34]. At the same time, lindenenol is a key differential metabolite identified by metabonomics (Table 1, non-volatile metabolites, number 24). The results of the quantitative analysis are presented in Table S11. Box plots were employed to graphically represent the content variations among the three root groups for these constituents (Fig. 3E). It was evident that the concentrations of these three compounds were markedly higher in the tuberous root group than in both the taproot and old root groups, while no significant differences were found between the taproot and old root groups. This can largely be attributed to the developmental stage and structure of tuberous roots. The cambium and extra-cambium activities during the development of the tuberous roots lead to expansion, with continuous division producing secondary tissue. This process contributes to the accumulation of secondary metabolites, such as alkaloids and terpenes, which are involved in the plant's defense and survival mechanisms [35].

##### 3.3.3.2 Quantification analysis of the three main indicators based on full wavelengths

After dividing the dataset into calibration and prediction sets, we developed BPNN models using various spectral pre-processing techniques for the calibration data. The optimal pre-processing was determined by minimizing the RMSEC through 10-fold cross-validation. The prediction set validated the model's accuracy on new data. The model utilized one-dimensional spectral data to predict the concentrations of *L. aggregata*'s three main constituents, as detailed in Table S12.

SNV pre-processing notably improved predictive accuracy for norisoboldine, linderane, and lindenenol, achieving $R_{p}^{2}$ values from 0.87 to 0.88, RMSEP values from 0.05 to 0.20, and RPD values from 2.88 to 2.93, highlighting the models’ strong predictive performance and the suitability of SNV pre-processing for further analyses.

##### 3.3.3.3 Quantification analysis of the main constituent using EWs

**Fig. 4:**
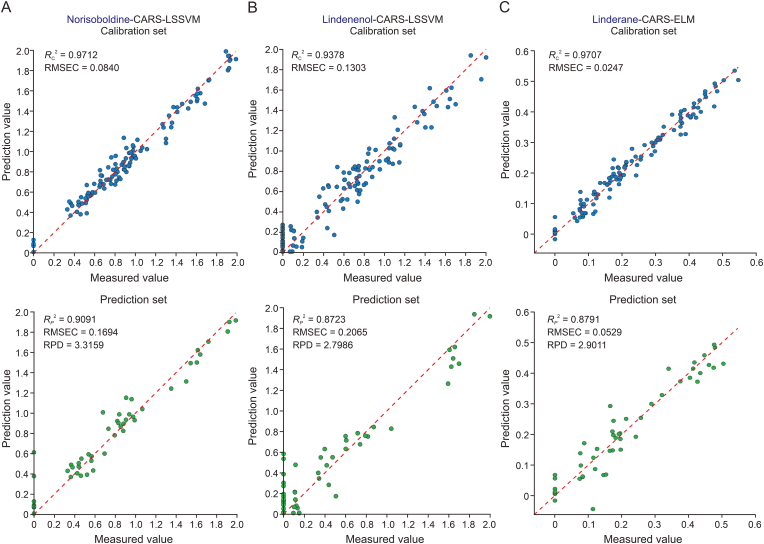
Scatter plots of the calibration and prediction results. (A) The calibration and prediction results of norisoboldine. (B) The calibration and prediction results of lindenenol. (C) The calibration and prediction results of linderane. $R_{c}^{2}$: coefficients of determination for calibration; RMSEC: root mean square error of calibration; $R_{P}^{2}$: coefficients of determination for prediction; RMSEP: root mean square error of prediction; RPD: ratio of prediction to deviation; LSSVM: least squares support vector machine; ELM: extreme learning machine; CARS: competitive adaptive reweighted sampling.

Spectral pre-processing enhances model prediction, but full-spectrum data can include irrelevant variables that impact model precision. Feature wavelength selection is employed to filter out the EWs from the entire spectrum to streamline the modeling process and enhance detection efficiency [14]. We applied CARS, SPA, and RF algorithms to select feature wavelengths, significantly reducing the number of variables by 80.37%–84.44%, 97.78%–99.26%, and 93.70%–94.07%, respectively (Fig. 3F). Model performance was assessed based on the *R*^2^ and RMSE of the test set. CARS was particularly effective, yielding the best predictive results with $R_{p}^{2}$ values ranging from 0.87 to 0.90, RMSEP between 0.05 and 0.20, and RPD from 2.79 to 3.31 (Table 4). However, for linderane and lindenenol, after variable selection using CARS, the model's performance is worse than that of the full-spectrum model. This may be because the CARS method removes essential variables during the selection process. To demonstrate the capacity of the LSSVM and ELM models more intuitively, scatter plots of calibration and prediction values of the norisoboldine, linderane, and lindenenol prediction models are presented in Fig. 4. The CARS-LSSVM model excelled for norisoboldine and lindenenol, achieving $R_{p}^{2}$ of 0.9091 and 0.8723, RMSEP of 0.1694 and 0.2065, and RPD of 3.3159 and 2.7986, respectively (Figs. 4A and B). The CARS-ELM model performed best for linderane, closely matching predictions to actual values ($R_{p}^{2}$ of 0.8791, RMSEP of 0.0529, and RPD of 2.9011) (Fig. 4C). There are no significant outliers in the scatterplots of Fig. 4, likely attributable to the homogenization effect of powdering all samples prior to analysis, which ensured compositional uniformity.

**Table 4:** Calibration and prediction results of different models based on full spectrum and different feature wavelength selection methods.

Constituents	Model	Selection method	EWs numbers	Calibration		Prediction		
				$R_{c}^{2}$	RMSEC	$R_{p}^{2}$	RMSEP	RPD
Norisoboldine	**LSSVM**	FS	270	0.9773	0.0711	0.7733	0.2926	2.1000
		**CARS**	**53**	**0.9712**	**0.0840**	**0.9091**	**0.1694**	**3.3159**
		SPA	2	0.2937	0.4096	0.1814	0.5086	1.1052
		RF	17	0.7665	0.2382	0.7854	0.2660	2.1584
	ELM	FS	270	0.0928	0.4495	0.0283	0.6057	1.0145
		CARS	53	0.8031	0.2194	0.7709	0.2688	2.0894
		SPA	2	0.4022	0.3768	0.2239	0.4952	1.1351
		RF	17	0.8003	0.2202	0.8117	0.2492	2.3042
	BPNN	FS	270	0.7347	0.2431	0.3863	0.4814	1.2765
		CARS	53	0.8956	0.1598	0.8400	0.2246	2.5003
		SPA	2	0.2697	0.4165	0.0338	0.5525	1.0174
		RF	17	0.8372	0.1989	0.8160	0.2463	2.3311
Linderane	LSSVM	FS	270	0.9993	0.0038	0.9042	0.0454	3.2305
		CARS	53	0.9239	0.0404	0.8571	0.0556	2.6456
		SPA	6	0.3002	0.1225	0.3879	0.1147	1.2782
		RF	16	0.7902	0.0677	0.6777	0.0828	1.7615
	**ELM**	FS	270	0.0506	0.1428	0.2312	0.1628	0.9012
		**CARS**	**53**	**0.9707**	**0.0247**	**0.8791**	**0.0529**	**2.9011**
		SPA	6	0.2718	0.0660	0.7965	0.1251	1.1719
		RF	16	0.9213	0.0415	0.6511	0.0861	1.6930
	BPNN	FS	270	0.6560	0.0860	0.4206	0.1117	1.3138
		CARS	53	0.8430	0.0580	0.8084	0.0644	2.2846
		SPA	6	0.5906	0.1093	0.4431	0.0938	1.5630
		RF	16	0.6990	0.0811	0.6783	0.0827	1.7631
Lindenenol	**LSSVM**	FS	270	0.9603	0.1005	0.9172	0.1809	3.4761
		**CARS**	**42**	**0.9378**	**0.1303**	**0.8723**	**0.2065**	**2.7986**
		SPA	6	0.5492	0.3530	0.4697	0.4176	1.3733
		RF	17	0.8655	0.1933	0.6948	0.3231	1.8101
	ELM	FS	270	0.1155	0.4744	0.0715	0.6059	1.0378
		CARS	42	0.8357	0.2118	0.7421	0.2935	1.9690
		SPA	6	0.7822	0.2046	0.0157	0.5690	1.0079
		RF	17	0.8519	0.2028	0.7674	0.2820	2.0736
	BPNN	FS	270	0.8726	0.1800	0.7553	0.3111	2.0214
		CARS	42	0.8677	0.1901	0.8297	0.2385	2.4230
		SPA	6	0.5135	0.3667	0.5003	0.4054	1.4146
		RF	17	0.8231	0.2217	0.7792	0.2748	2.1282

EWs: effective wavelengths; $R_{c}^{2}$: coefficients of determination for calibration; RMSEC: root mean square error of calibration; $R_{P}^{2}$: coefficients of determination for prediction; RMSEP: root mean square error of prediction; RPD: ratio of prediction to deviation; LSSVM: least squares support vector machine; ELM: extreme learning machine; BPNN: backpropagation neural network; FS: full spectrum; CARS: competitive adaptive reweighted sampling; SPA: successive projections algorithm; RF:random frog.

### 3.4 Visualization of the typical marker compound norisoboldine and merge in slices

**Fig. 5:**
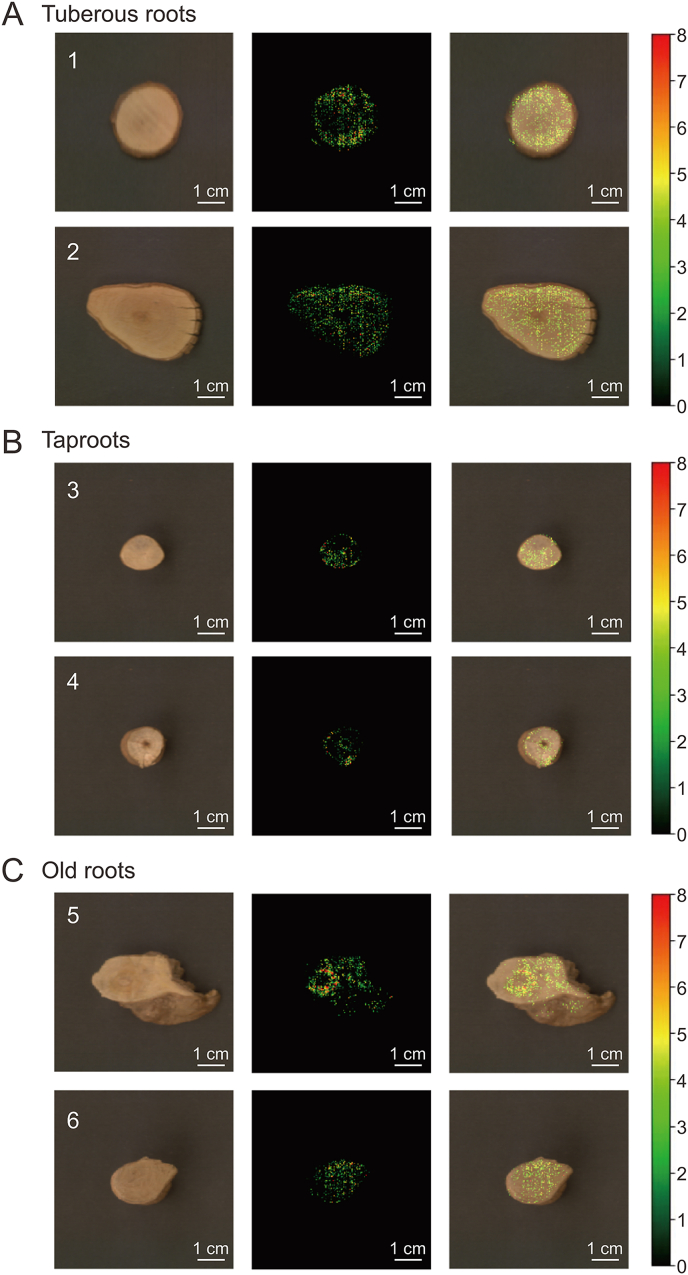
Visualized norisboldine distribution of slices in three root types of *L. aggregata*. The gradient bar chart represents the content of norisboldine in this area. (A) The distribution images of tuberous root's slice. (B) The distribution images of taproot's slice. (C) The distribution images of old root's slice.

HSI offers a unique combination of spectral and spatial information, facilitating the direct visualization of sample component distribution. Norisoboldine, a typical indicator in Linderae Radix, was chosen for visualization due to its superior predictive performance, with an $R_{p}^{2}$ of 0.9091 and an RPD value above 3, indicating high goodness-of-fit and predictive accuracy. Each pixel within the hyperspectral data captured a reflection spectrum, which was utilized to characterize the physiological and color parameters of *L. aggregata* across different root types. The SNV-CARS-LSSVM model, optimized for wavelength selection, was applied to predict norisoboldine distribution across *L. aggregata* root samples. This model's predictions were then mapped onto each hyperspectral image pixel to create a visualization, as seen in Fig. 5, where colors transition from red (high concentration) to black (low concentration).

Fig. 5 illustrate a more extensive distribution in tuberous root slices (Fig. 5A) compared to taproot slices and old root slices (Figs. 5B and C), with a higher number of visible pixel points. The taproot slices, especially compared with tuberous root slices and old root slices, have only pixels at the edge. This visualization aligns with the quantitative data in Fig. 3E, marking the first application of HSI to predict norisoboldine content in various *L. aggregata* root types. The color maps visually assess component variation and distribution, offering valuable insights into the quality of different root types of *L. aggregata*.

## 4 Conclusion and limitations

This study integrates VIS-NIR HIS with non-targeted metabolomics to investigate the metabolic differences and predict the chemical composition of *L. aggregata* across its various root types. By employing plant metabolomics analysis using UPLC-QTOF-MS and GC-MS, we identified 25 and 48 differential metabolites in the alcoholic extracts and volatile oils, respectively, which provide an orientation for quality assessment of Linderae Radix. Moreover, a typical differentiated compound together with quality indicators documented in Chinese Pharmacopoeia (2020), were chosen as indicators for quantitative analysis and combined with HSI for classification, achieving 93.33% classification accuracy with 2^nd^D-SG-LR models. Furthermore, through SNV pre-processing, feature wavelengths were effectively selected using methods such as CARS, SPA, and RF, and the SNV-CARS-LSSVM and SNV-CARS-ELM models demonstrated high predictive accuracy for significant chemical constituents. The ability to visualize the spatial distribution of norisoboldine content across different root types through pixel mapping provides an innovative, non-invasive method to assess the chemical profile of *L. aggregata*.

This study presents a pioneering, comprehensive approach that integrates metabolomics with HSI to classify, quantify, and visualize spectral information and key chemical constituents in *L. aggregata*, thereby establishing a rapid and non-destructive method for quality assessment. The findings lay a solid scientific foundation for facilitating the sustainable utilization of *L. aggregata* in the market through standardized quality evaluation. Nevertheless, the research has certain limitations. Notably, it relied primarily on root samples from limited geographic regions and batches, which may compromise the robustness of the developed models. Future research focused on practical quality assessment applications should prioritize incorporating a broader range of variables (such as ecological conditions and cultivation practices) and addressing issues like sensitivity to particle size and moisture content. These advancements will be crucial for developing a more comprehensive and reliable model—one that can be universally applied to quality control of *L. aggregata* and potentially extended to other medicinal plants.

## CRediT authorship contribution statement

**Yangbin Lv:** Writing – original draft, Validation, Methodology, Data curation, Conceptualization. **Hongwei Sun:** Methodology, Data curation. **Qiaoling Ding:** Methodology. **Bangxu Chen:** Visualization. **Hongwei Ye:** Methodology, Data curation. **Ning Xu:** Writing – review & editing, Methodology, Funding acquisition, Conceptualization. **Chu Chu:** Writing – review & editing, Resources, Project administration, Methodology, Funding acquisition, Conceptualization.

## Declaration of competing interest

The authors declare that they have no known competing financial interests or personal relationships that could have appeared to influence the work reported in this paper.
