# Supplementary material for: Authentication of Linderae Radix through plant metabolomics coupled with a machine learning-enhanced in situ hyperspectral imaging approach
Source: J Pharm Anal. 2025 Oct 28;16(5):101476. doi: 10.1016/j.jpha.2025.101476 (PMC13213644; doi:10.1016/j.jpha.2025.101476)
Supplement: Multimedia component 1 [file mmc1.docx]

**SUPPLEMENTARY MATERIAL** to the paper entitled:

**Table S1** Samples collecting information of *L. aggregata.*

**Table S2** Linear regression equations, range, limit of detection, and limit of quantification for three chemical components.

**Table S3** Intra-day precision results of the 3 quantitative assay markers (*n* = 6)

**Table S4** Inter-day precision results of the 3 quantitative constituents (*n* = 3)

**Table S5** Stability results of the 3 quantitative constituents.

**Table S6** Repeatability results of the 3 quantitative constituents (*n* = 6).

**Table S7** Recovery experiment results of the 3 quantitative constituents (*n* = 6)

**Table S8** List of all identified metabolites of 49 batches of *L. aggregata* samples by ultra-performance liquid chromatography-quadrupole time-of-flight mass spectrometry (UPLC-QTOF-MS).

**Table S9** Cross-validation based on the corresponding partial least squares discrimination analysis (PLS-DA) model.

**Table S10** List of all identified metabolites of 49 batches of *L. aggregata* samples by gas chromatography-mass spectrometry (GC-MS).

**Table S11** Contents of index components in the *L. aggregata* samples.

**Table S12** Performances of backpropagation neural network (BPNN) models with different pre-processing for predicting index constituents of *L. aggregata*.

**Fig. S1** The total ion current (TIC) overlapping map in positive ion mode by ultra-performance liquid chromatography-quadrupole time-of-flight mass spectrometry (UPLC-QTOF-MS) of three root types of *L. aggregata* samples, including quality control (QC) sample results. Note: The abscissa is the acquisition time of the metabolite. The ordinate is the ion current intensity of the ion detection. QC: quality control.

**Fig. S2** Cross-validation based on the corresponding (partial least squares discrimination analysis) PLS-DA model based on ultra-performance liquid chromatography-quadrupole time-of-flight mass spectrometry (UPLC-QTOF-MS) metabolomics data. The model’s predictive power can be reflected in the magnitude of the Q^2^ value, while R^2^ represents the total variance the model can explain.

**Fig. S3** Heatmaps of hierarchical cluster analysis (HCA) by ultra-performance liquid chromatography-quadrupole time-of-flight mass spectrometry (UPLC-QTOF-MS) in three root types of *L. aggregata*. (A) The heatmap of HCA of total differential metabolites. (B) The heatmap of HCA of 25 differential metabolites.

**Fig. S4** The total ion current (TIC) overlapping map in positive ion mode by gas chromatography-mass spectrometry (GC-MS) of three root types of *L. aggregata* samples, including quality control (QC) sample results. Note: The abscissa is the acquisition time of the metabolite. The ordinate is the ion current intensity of the ion detection. QC: quality control.

**Fig. S5** Cross-validation based on the corresponding (partial least squares discrimination analysis) PLS-DA model based on gas chromatography-mass spectrometry (GC-MS) metabolomics data. The model’s predictive power can be reflected in the magnitude of the Q2 value, while R2 represents the total variance the model can explain.

**Fig. S6** Heatmaps of hierarchical cluster analysis (HCA) by gas chromatography-mass spectrometry (GC-MS) in three root types of *L. aggregata*. (A) The heatmap of HCA of total differential metabolites. (B) The heatmap of HCA of 48 differential metabolites.

**Fig.S7** Hyperspectral images of all samples.

**Table S1** Samples collecting information of *L. aggregata.*

| No. | Sample Name | [Origin](javascript:%20void(0)) | Longitudes | Latitude | Growth pattern | Root type |
| --- | --- | --- | --- | --- | --- | --- |
| 1 | K1 | Zhengjiang Quzhou | 28.97 | 118.86 | Wild cultivation | Tuberous roots |
| 2 | K2 | Zhengjiang Quzhou | 28.97 | 118.86 | Wild cultivation | Tuberous roots |
| 3 | K3 | Zhengjiang Quzhou | 28.97 | 118.86 | Wild cultivation | Tuberous roots |
| 4 | K4 | Zhengjiang Quzhou | 28.97 | 118.86 | Wild cultivation | Tuberous roots |
| 5 | K5 | Zhengjiang Quzhou | 28.97 | 118.86 | Wild cultivation | Tuberous roots |
| 6 | K6 | Zhengjiang Quzhou | 28.97 | 118.86 | Wild cultivation | Tuberous roots |
| 7 | K7 | Zhengjiang Hangzhou | 29.47 | 119.28 | Artificial cultivation | Tuberous roots |
| 8 | K8 | Zhengjiang Hangzhou | 29.47 | 119.28 | Artificial cultivation | Tuberous roots |
| 9 | K9 | Zhengjiang  Taizhou | 28.66 | 121.42 | Artificial cultivation | Tuberous roots |
| 10 | K10 | Zhengjiang  Taizhou | 28.66 | 121.42 | Artificial cultivation | Tuberous roots |
| 11 | K11 | Zhengjiang  Taizhou | 28.66 | 121.42 | Artificial cultivation | Tuberous roots |
| 12 | K12 | Zhengjiang | 29.14 | 121.01 | Artificial cultivation | Tuberous roots |
| 13 | K13 | Tiantai | 29.14 | 121.01 | Artificial cultivation | Tuberous roots |
| 14 | K14 | Zhengjiang | 28.45 | 117.94 | Wild cultivation | Tuberous roots |
| 15 | K15 | Tiantai | 28.45 | 117.94 | Wild cultivation | Tuberous roots |
| 16 | K16 | Jiangxi Shangrao | 28.45 | 117.94 | Wild cultivation | Tuberous roots |
| 17 | K17 | Jiangxi Shangrao | 28.45 | 117.94 | Wild cultivation | Tuberous roots |
| 18 | K18 | Jiangxi Shangrao | 28.45 | 117.94 | Wild cultivation | Tuberous roots |
| 19 | K19 | Jiangxi Shangrao | 28.45 | 117.94 | Wild cultivation | Tuberous roots |
| 20 | K20 | Jiangxi Shangrao | 28.45 | 117.94 | Wild cultivation | Tuberous roots |
| 21 | K21 | Jiangxi Shangrao | 28.45 | 117.94 | Wild cultivation | Tuberous roots |
| 22 | K22 | Jiangxi Shangrao | 28.45 | 117.94 | Wild cultivation | Tuberous roots |
| 23 | K23 | Jiangxi Shangrao | 28.45 | 117.94 | Wild cultivation | Tuberous roots |
| 24 | K24 | Jiangxi Shangrao | 28.45 | 117.94 | Wild cultivation | Tuberous roots |
| 25 | K25 | Jiangxi Shangrao | 28.45 | 117.94 | Wild cultivation | Tuberous roots |
| 26 | K26 | Jiangxi Shangrao | 28.45 | 117.94 | Wild cultivation | Tuberous roots |
| 27 | K27 | Jiangxi Jiujiang | 29.70 | 116.00 | Wild cultivation | Tuberous roots |
| 28 | K28 | Hunan  Yongzhou | 26.42 | 111.61 | Artificial cultivation | Tuberous roots |
| 29 | K29 | Hunan  Yongzhou | 26.42 | 111.61 | Artificial cultivation | Tuberous roots |
| 30 | L1 | Hunan  Loudi | 27.70 | 111.99 | Wild cultivation | Old roots |
| 31 | L2 | Hunan | 27.70 | 111.99 | Wild cultivation | Old roots |
| 32 | L3 | Loudi | 27.70 | 111.99 | Wild cultivation | Old roots |
| 33 | L4 | Hunan | 27.70 | 111.99 | Wild cultivation | Old roots |
| 34 | L5 | Loudi | 27.70 | 111.99 | Wild cultivation | Old roots |
| 35 | L6 | Hunan | 27.70 | 111.99 | Wild cultivation | Old roots |
| 36 | L7 | Loudi | 27.70 | 111.99 | Wild cultivation | Old roots |
| 37 | L8 | Hunan | 27.70 | 111.99 | Wild cultivation | Old roots |
| 38 | L9 | Loudi | 27.70 | 111.99 | Wild cultivation | Old roots |
| 39 | L10 | Hunan | 27.70 | 111.99 | Wild cultivation | Old roots |
| 40 | Z1 | Guangdong  Shaoguan | 24.81 | 113.60 | Wild cultivation | Taproots |
| 41 | Z2 | Guangdong | 24.81 | 113.60 | Wild cultivation | Taproots |
| 42 | Z3 | Shaoguan | 24.81 | 113.60 | Wild cultivation | Taproots |
| 43 | Z4 | Guangdong | 24.81 | 113.60 | Wild cultivation | Taproots |
| 44 | Z5 | Shaoguan | 24.81 | 113.60 | Wild cultivation | Taproots |
| 45 | Z6 | Guangdong | 24.81 | 113.60 | Wild cultivation | Taproots |
| 46 | Z7 | Shaoguan | 24.81 | 113.60 | Wild cultivation | Taproots |
| 47 | Z8 | Guangdong | 24.81 | 113.60 | Wild cultivation | Taproots |
| 48 | Z9 | Shaoguan | 24.81 | 113.60 | Wild cultivation | Taproots |
| 49 | Z10 | Guangdong | 24.81 | 113.60 | Wild cultivation | Taproots |

**Table S2** Linear regression equations, range, limit of detection and limit of quantification for three chemical components.

| No. | [Constituent](javascript:%20void(0)) | Standard curve | R^2^ | linear range (μg/mL) | LOD  (μg/mL) | LOQ  (μg/mL) |
| --- | --- | --- | --- | --- | --- | --- |
| 1 | Norisoboldine | Y=32.958X-323.61 | 0.9993 | 25-600 | 3.6 | 12 |
| 2 | Linderane | Y=13.52X-44.183 | 0.9993 | 25-600 | 2.9 | 9.6 |
| 3 | Lindenenol | Y=7.3782X+7.7775 | 0.9995 | 25-600 | 3.2 | 10.6 |

LOD: limit of quantitation; LOQ: limit of quantitation.

**Table S3** Intra-day precision results of the 3 quantitative assay markers (*n* = 6).

| No. | Retention time (min) | RSD (%) | Content (mg/g) | RSD (%) |
| --- | --- | --- | --- | --- |
| Norisoboldine | | | | |
| 1 | 14.173 | 0.49 | 5.8502 | 1.98 |
| 2 | 14.252 |  | 5.8454 |  |
| 3 | 14.357 |  | 5.6680 |  |
| 4 | 14.291 |  | 5.7976 |  |
| 5 | 14.217 |  | 5.8383 |  |
| 6 | 14.184 |  | 6.0166 |  |
| Linderane | | | | |
| 1 | 28.427 | 0.08 | 1.9985 | 1.18 |
| 2 | 28.391 |  | 2.0350 |  |
| 3 | 28.447 |  | 2.0069 |  |
| 4 | 28.457 |  | 1.9910 |  |
| 5 | 28.429 |  | 1.9680 |  |
| 6 | 28.413 |  | 2.0168 |  |
| Lindenenol | | | | |
| 1 | 29.697 | 0.13 | 3.8983 | 1.53 |
| 2 | 29.598 |  | 3.9346 |  |
| 3 | 29.693 |  | 4.0037 |  |
| 4 | 29.674 |  | 4.0151 |  |
| 5 | 29.653 |  | 3.9035 |  |
| 6 | 29.641 |  | 4.0384 |  |

RSD: relative standard deviation.

**Table S4** Inter-day precision results of the 3 quantitative constituents (*n* = 3).

| No. | Retention time (min) | RSD (%) | Content (mg/g) | RSD (%) |
| --- | --- | --- | --- | --- |
| Norisoboldine | | | | |
| Day-1-1 | 14.374 | 0.57 | 5.8234 | 2.58 |
| Day-1-2 | 14.176 |  | 5.8367 |  |
| Day-1-3 | 14.299 |  | 5.8527 |  |
| Day-2-1 | 14.265 |  | 6.1749 |  |
| Day-2-2 | 14.366 |  | 5.9501 |  |
| Day-2-3 | 14.373 |  | 5.7047 |  |
| Day-3-1 | 14.173 |  | 5.8502 |  |
| Day-3-2 | 14.252 |  | 5.8454 |  |
| Day-3-3 | 14.357 |  | 5.6680 |  |
| Linderane | | | | |
| Day-1-1 | 28.354 | 0.15 | 1.9855 | 1.94 |
| Day-1-2 | 28.364 |  | 2.0602 |  |
| Day-1-3 | 28.419 |  | 2.0160 |  |
| Day-2-1 | 28.384 |  | 1.9806 |  |
| Day-2-2 | 28.439 |  | 2.0855 |  |
| Day-2-3 | 28.317 |  | 1.9736 |  |
| Day-3-1 | 28.427 |  | 1.9985 |  |
| Day-3-2 | 28.391 |  | 2.0350 |  |
| Day-3-3 | 28.447 |  | 2.0069 |  |
| Lindenenol | | | | |
| Day-1-1 | 29.594 | 0.16 | 3.6671 | 4.08 |
| Day-1-2 | 29.640 |  | 3.5971 |  |
| Day-1-3 | 29.641 |  | 3.7874 |  |
| Day-2-1 | 29.656 |  | 3.7007 |  |
| Day-2-2 | 29.693 |  | 3.5996 |  |
| Day-2-3 | 29.574 |  | 3.6321 |  |
| Day-3-1 | 29.697 |  | 3.8983 |  |
| Day-3-2 | 29.598 |  | 3.9346 |  |
| Day-3-3 | 29.693 |  | 4.0037 |  |

RSD: relative standard deviation.

**Table S5** Stability results of the 3 quantitative constituents.

| Time (h) | Retention time (min) | RSD (%) | Content (mg/g) | RSD (%) |
| --- | --- | --- | --- | --- |
| Norisoboldine | | | | |
| 0 | 14.117 | 2.65 | 5.3255 | 4.38 |
| 2 | 13.293 |  | 5.7420 |  |
| 4 | 14.357 |  | 5.2620 |  |
| 6 | 14.168 |  | 5.5487 |  |
| 12 | 13.875 |  | 5.8321 |  |
| 24 | 13.891 |  | 5.7235 |  |
| Linderane | | | | |
| 0 | 28.561 | 0.41 | 1.9168 | 3.36 |
| 2 | 28.271 |  | 1.9836 |  |
| 4 | 28.330 |  | 1.8263 |  |
| 6 | 28.416 |  | 1.8688 |  |
| 12 | 28.301 |  | 1.9059 |  |
| 24 | 28.504 |  | 1.9822 |  |
| Lindenenol | | | | |
| 0 | 29.820 | 0.41 | 3.9371 | 3.95 |
| 2 | 29.585 |  | 4.2575 |  |
| 4 | 29.566 |  | 3.9116 |  |
| 6 | 29.662 |  | 3.7874 |  |
| 12 | 29.526 |  | 4.0542 |  |
| 24 | 29.780 |  | 4.0084 |  |

RSD: relative standard deviation.

**Table S6** Repeatability results of the 3 quantitative constituents (*n* = 6).

| No. | Retention time (min) | RSD (%) | Content (mg/g) | RSD (%) |
| --- | --- | --- | --- | --- |
| Norisoboldine | | | | |
| 1 | 13.703 | 2.35 | 5.5678 | 1.89 |
| 2 | 13.688 |  | 5.6508 |  |
| 3 | 13.568 |  | 5.4304 |  |
| 4 | 13.479 |  | 5.4533 |  |
| 5 | 14.136 |  | 5.3857 |  |
| 6 | 14.286 |  | 5.5587 |  |
| Linderane | | | | |
| 1 | 28.001 | 0.28 | 2.1044 | 3.25 |
| 2 | 28.014 |  | 1.9608 |  |
| 3 | 27.971 |  | 2.0944 |  |
| 4 | 28.067 |  | 2.0519 |  |
| 5 | 28.086 |  | 1.9815 |  |
| 6 | 28.023 |  | 2.1066 |  |
| Lindenenol | | | | |
| 1 | 28.475 | 0.48 | 4.3139 | 4.73 |
| 2 | 28.479 |  | 4.2705 |  |
| 3 | 28.422 |  | 4.5486 |  |
| 4 | 28.639 |  | 3.9463 |  |
| 5 | 28.593 |  | 4.3328 |  |
| 6 | 28.513 |  | 4.4467 |  |

RSD: relative standard deviation.

**Table S7** Recovery experiment results of the 3 quantitative constituents (*n* = 6).

| Compound | No. | Sample weight (g) | Original amount (mg) | Added amount (mg) | Found amount (mg) | Recovery rate  (%) | Average  recovery （%） | RSD (%) |  |
| --- | --- | --- | --- | --- | --- | --- | --- | --- | --- |
| Mixed solution with each quantitative marker concentration of 25 μg/mL | | | | | | | | | |
| Norisoboldine | 1 | 0.1995 | 1.0988 | 0.0250 | 1.1650 | 103.66 | 97.35 | 4.89 |  |
|  | 2 | 0.2004 | 1.1038 | 0.0250 | 1.0872 | 96.32 |  |  |  |
|  | 3 | 0.2006 | 1.1049 | 0.0250 | 1.0876 | 96.26 |  |  |  |
|  | 4 | 0.2007 | 1.1054 | 0.0250 | 1.1612 | 102.72 |  |  |  |
|  | 5 | 0.2002 | 1.1027 | 0.0250 | 1.0364 | 91.90 |  |  |  |
|  | 6 | 0.1993 | 1.0977 | 0.0250 | 1.0470 | 93.25 |  |  |  |
| Linderane | 1 | 0.1995 | 0.4090 | 0.0250 | 0.3752 | 86.47 | 85.38 | 3.92 |  |
|  | 2 | 0.2004 | 0.4108 | 0.0250 | 0.3971 | 91.12 |  |  |  |
|  | 3 | 0.2006 | 0.4112 | 0.0250 | 0.3682 | 84.41 |  |  |  |
|  | 4 | 0.2007 | 0.4114 | 0.0250 | 0.3743 | 85.76 |  |  |  |
|  | 5 | 0.2002 | 0.4104 | 0.0250 | 0.3620 | 83.15 |  |  |  |
|  | 6 | 0.1993 | 0.4086 | 0.0250 | 0.3529 | 81.40 |  |  |  |
| Lindenenol | 1 | 0.1995 | 0.8598 | 0.0250 | 0.8265 | 93.42 | 90.04 | 2.32 |  |
|  | 2 | 0.2004 | 0.8637 | 0.0250 | 0.8098 | 91.12 |  |  |  |
|  | 3 | 0.2006 | 0.8645 | 0.0250 | 0.7755 | 87.18 |  |  |  |
|  | 4 | 0.2007 | 0.8650 | 0.0250 | 0.7942 | 89.24 |  |  |  |
|  | 5 | 0.2002 | 0.8628 | 0.0250 | 0.7944 | 89.48 |  |  |  |
|  | 6 | 0.1993 | 0.8589 | 0.0250 | 0.7939 | 89.82 |  |  |  |
| Mixed solution with each quantitative marker concentration of 200 μg/mL | | | | | | | | | |
| Norisoboldine | 1 | 0.2005 | 1.1043 | 0.2000 | 1.3360 | 102.43 | 101.30 | 1.44 |  |
|  | 2 | 0.2013 | 1.1087 | 0.2000 | 1.2959 | 99.02 |  |  |  |
|  | 3 | 0.1998 | 1.1005 | 0.2000 | 1.3043 | 100.30 |  |  |  |
|  | 4 | 0.1994 | 1.0983 | 0.2000 | 1.3244 | 102.02 |  |  |  |
|  | 5 | 0.2006 | 1.1049 | 0.2000 | 1.3193 | 101.11 |  |  |  |
|  | 6 | 0.1993 | 1.0977 | 0.2000 | 1.3356 | 102.92 |  |  |  |
| Linderane | 1 | 0.2005 | 0.4110 | 0.2000 | 0.6308 | 103.24 | 102.73 | 2.59 |  |
|  | 2 | 0.2013 | 0.4127 | 0.2000 | 0.5987 | 97.72 |  |  |  |
|  | 3 | 0.1998 | 0.4096 | 0.2000 | 0.6392 | 104.86 |  |  |  |
|  | 4 | 0.1994 | 0.4088 | 0.2000 | 0.6217 | 102.13 |  |  |  |
|  | 5 | 0.2006 | 0.4112 | 0.2000 | 0.6338 | 103.69 |  |  |  |
|  | 6 | 0.1993 | 0.4086 | 0.2000 | 0.6376 | 104.77 |  |  |  |
| Lindenenol | 1 | 0.2005 | 0.8641 | 0.2000 | 1.0981 | 103.19 | 102.10 | 1.30 |  |
|  | 2 | 0.2013 | 0.8676 | 0.2000 | 1.0623 | 99.50 |  |  |  |
|  | 3 | 0.1998 | 0.8611 | 0.2000 | 1.0840 | 102.16 |  |  |  |
|  | 4 | 0.1994 | 0.8594 | 0.2000 | 1.0829 | 102.22 |  |  |  |
|  | 5 | 0.2006 | 0.8645 | 0.2000 | 1.0932 | 102.69 |  |  |  |
|  | 6 | 0.1993 | 0.8589 | 0.2000 | 1.0889 | 102.83 |  |  |  |
| Mixed solution with each quantitative marker concentration of 500 μg/mL | | | | | | | | | |
| Norisoboldine | 1 | 0.1994 | 1.0983 | 0.5000 | 1.7468 | 109.29 | 103.44 | 2.88 |  |
|  | 2 | 0.2005 | 1.1043 | 0.5000 | 1.6432 | 102.42 |  |  |  |
|  | 3 | 0.2002 | 1.1027 | 0.5000 | 1.6476 | 102.80 |  |  |  |
|  | 4 | 0.1998 | 1.1005 | 0.5000 | 1.6158 | 100.96 |  |  |  |
|  | 5 | 0.1997 | 1.0999 | 0.5000 | 1.6529 | 103.32 |  |  |  |
|  | 6 | 0.1999 | 1.1010 | 0.5000 | 1.6305 | 101.84 |  |  |  |
| Linderane | 1 | 0.1994 | 0.4088 | 0.5000 | 0.8163 | 89.83 | 93.21 | 1.97 |  |
|  | 2 | 0.2005 | 0.4110 | 0.5000 | 0.8595 | 94.35 |  |  |  |
|  | 3 | 0.2002 | 0.4104 | 0.5000 | 0.8554 | 93.96 |  |  |  |
|  | 4 | 0.1998 | 0.4096 | 0.5000 | 0.8633 | 94.91 |  |  |  |
|  | 5 | 0.1997 | 0.4094 | 0.5000 | 0.8416 | 92.55 |  |  |  |
|  | 6 | 0.1999 | 0.4098 | 0.5000 | 0.8522 | 93.67 |  |  |  |
| Lindenenol | 1 | 0.1994 | 0.8594 | 0.5000 | 1.3712 | 100.87 | 97.52 | 3.90 |  |
|  | 2 | 0.2005 | 0.8641 | 0.5000 | 1.3489 | 98.89 |  |  |  |
|  | 3 | 0.2002 | 0.8628 | 0.5000 | 1.3619 | 99.93 |  |  |  |
|  | 4 | 0.1998 | 0.8611 | 0.5000 | 1.3235 | 97.23 |  |  |  |
|  | 5 | 0.1997 | 0.8607 | 0.5000 | 1.3331 | 97.98 |  |  |  |
|  | 6 | 0.1999 | 0.8615 | 0.5000 | 1.2285 | 90.23 |  |  |  |

RSD: relative standard deviation.

**Table S8** List of all identified metabolites of 49 batches of *L. aggregata* samples by ultra-performance liquid chromatography-quadrupole time-of-flight mass spectrometry (UPLC-QTOF-MS).

The data for this table are available in the supplementary Excel file.

**Table S9** Cross-validation based on the corresponding partial least squares discrimination analysis (PLS-DA) model.

| Measure | UPLC-QTOF-MS | | | GC-MS | |
| --- | --- | --- | --- | --- | --- |
| Number of compounds | 1 | 2 | 3 | 1 | 2 |
| Accuracy (%) | 79.592 | 100.000 | 100.000 | 75.510 | 100.000 |
| R^2^X | 0.190 | 0.298 | 0.561 | 0.298 | 0.402 |
| R^2^Y | 0.531 | 0.916 | 0.993 | 0.413 | 0.795 |
| Q^2^Y | 0.479 | 0.717 | 0.848 | 0.401 | 0.577 |

UPLC-QTOF-MS: ultra-performance liquid chromatography-quadrupole time-of-flight mass spectrometry; GC-MS: gas chromatography-mass spectrometry.

**Table S10** List of all identified metabolites of 49 batches of *L. aggregata* samples by gas chromatography-mass spectrometry (GC-MS).

The data for this table are available in the supplementary Excel file.

**Table S11** Contents of index components in the *L. aggregata* samples*.*

| SampleNo. | Sample Name | Norisoboldine  （mg/g） | Linderane  （mg/g） | Lindenenol  （mg/g） | SampleNo. | Sample Name | Norisoboldine  （mg/g） | Linderane  （mg/g） | Lindenenol  （mg/g） |
| --- | --- | --- | --- | --- | --- | --- | --- | --- | --- |
| 1 | K1 (1) | 3.2625 | 0.8606 | 2.1960 | 74 | K25 (2) | 10.7769 | 1.8723 | 2.8601 |
| 2 | K1 (2) | 3.3960 | 0.8640 | 2.2662 | 75 | K25 (3) | 10.5505 | 1.8799 | 2.6633 |
| 3 | K1 (3) | 3.2444 | 0.8121 | 2.2377 | 76 | K26 (1) | 3.3018 | 1.4399 | 2.9436 |
| 4 | K2 (1) | 4.0059 | 1.8688 | 5.4120 | 77 | K26 (2) | 3.3696 | 1.5383 | 3.0545 |
| 5 | K2 (2) | 4.2005 | 1.7751 | 5.7771 | 78 | K26 (3) | 3.4368 | 1.5444 | 3.1754 |
| 6 | K2 (3) | 4.3276 | 1.8809 | 5.4941 | 79 | K27 (1) | 4.4730 | 1.4643 | 5.2181 |
| 7 | K3 (1) | 4.7725 | 1.4961 | 5.2347 | 80 | K27 (2) | 4.8057 | 1.5716 | 5.0783 |
| 8 | K3 (2) | 4.8828 | 1.6013 | 5.2526 | 81 | K27 (3) | 4.7417 | 1.6093 | 4.7766 |
| 9 | K3 (3) | 5.0992 | 1.6228 | 5.5036 | 82 | K28 (1) | 7.2924 | 2.0881 | 2.1895 |
| 10 | K4 (1) | 3.2864 | 0.8090 | 3.7381 | 83 | K28 (2) | 8.0384 | 2.1581 | 2.2708 |
| 11 | K4 (2) | 4.0077 | 0.9846 | 4.7570 | 84 | K28 (3) | 8.0398 | 1.9827 | 2.1738 |
| 12 | K4 (3) | 3.4712 | 0.9553 | 4.4179 | 85 | K29 (1) | 9.1969 | 2.7356 | 3.2456 |
| 13 | K5 (1) | 4.3957 | 0.8457 | 3.6608 | 86 | K29 (2) | 9.4427 | 2.5220 | 3.0046 |
| 14 | K5 (2) | 4.0675 | 0.9100 | 3.6600 | 87 | K29 (3) | 9.5973 | 2.6810 | 2.9745 |
| 15 | K5 (3) | 4.0832 | 0.8284 | 3.7281 | 88 | L1 (1) | 4.1023 | 0.9072 | 0.7572 |
| 16 | K6 (1) | 3.9076 | 1.0452 | 3.8555 | 89 | L1 (2) | 4.4312 | 0.9393 | 0.7588 |
| 17 | K6 (2) | 3.8592 | 1.0541 | 3.9962 | 90 | L1 (3) | 4.0313 | 0.9204 | 0.7197 |
| 18 | K6 (3) | 3.7650 | 0.9763 | 3.7039 | 91 | L2 (1) | 3.3588 | 0.4160 | 0.0000 |
| 19 | K7 (1) | 5.5678 | 2.1044 | 3.9579 | 92 | L2 (2) | 3.5462 | 0.4291 | 0.0000 |
| 20 | K7 (2) | 5.5150 | 2.0745 | 4.0883 | 93 | L2 (3) | 3.6749 | 0.4302 | 0.0000 |
| 21 | K7 (3) | 5.3462 | 2.1012 | 4.0455 | 94 | L3 (1) | 6.3293 | 0.5858 | 0.0000 |
| 22 | K8 (1) | 9.5536 | 2.4198 | 5.4122 | 95 | L3 (2) | 6.4988 | 0.4572 | 0.0000 |
| 23 | K8 (2) | 9.5960 | 2.3063 | 5.7302 | 96 | L3 (3) | 6.3793 | 0.4412 | 0.0000 |
| 24 | K8 (3) | 9.6519 | 2.2519 | 5.7733 | 97 | L4 (1) | 2.6116 | 0.3302 | 0.0000 |
| 25 | K9 (1) | 7.0516 | 1.8948 | 3.5494 | 98 | L4 (2) | 2.6130 | 0.2725 | 0.0000 |
| 26 | K9 (2) | 7.5921 | 2.0244 | 3.5906 | 99 | L4 (3) | 2.6681 | 0.2954 | 0.0000 |
| 27 | K9 (3) | 7.0510 | 2.0642 | 3.6112 | 100 | L5 (1) | 2.7536 | 1.0356 | 0.9491 |
| 28 | K10 (1) | 8.5057 | 2.5177 | 3.6844 | 101 | L5 (2) | 2.5537 | 0.9757 | 1.0133 |
| 29 | K10 (2) | 6.7949 | 2.1334 | 3.7731 | 102 | L5 (3) | 2.5644 | 0.9664 | 0.9675 |
| 30 | K10 (3) | 6.7559 | 2.0214 | 1.9946 | 103 | L6 (1) | 4.8736 | 0.5717 | 0.0000 |
| 31 | K11 (1) | 7.4952 | 1.4442 | 1.8032 | 104 | L6 (2) | 4.6544 | 0.5725 | 0.0000 |
| 32 | K11 (2) | 8.5859 | 1.6611 | 2.0914 | 105 | L6 (3) | 4.5557 | 0.5779 | 0.0000 |
| 33 | K11 (3) | 8.1856 | 1.2492 | 2.5348 | 106 | L7 (1) | 5.0216 | 0.3809 | 0.3595 |
| 34 | K12 (1) | 4.6911 | 2.3964 | 8.0386 | 107 | L7 (2) | 4.9561 | 0.4026 | 0.3974 |
| 35 | K12 (2) | 4.5310 | 2.3759 | 8.1224 | 108 | L7 (3) | 4.6951 | 0.4765 | 0.4251 |
| 36 | K12 (3) | 4.8847 | 2.3837 | 8.2153 | 109 | L8 (1) | 8.6186 | 1.9488 | 4.0853 |
| 37 | K13 (1) | 2.6110 | 2.0696 | 9.2405 | 110 | L8 (2) | 8.4097 | 1.8723 | 4.2062 |
| 38 | K13 (2) | 2.6892 | 2.1759 | 9.7599 | 111 | L8 (3) | 7.9521 | 1.9328 | 4.3984 |
| 39 | K13 (3) | 2.8400 | 2.2383 | 9.9944 | 112 | L9 (1) | 1.7567 | 0.0000 | 0.0000 |
| 40 | K14 (1) | 4.4417 | 1.0637 | 3.0011 | 113 | L9 (2) | 1.7755 | 0.0000 | 0.0000 |
| 41 | K14 (2) | 5.2555 | 1.3367 | 2.6869 | 114 | L9 (3) | 2.3858 | 0.0000 | 0.0000 |
| 42 | K14 (3) | 4.9651 | 1.2032 | 3.2773 | 115 | L10 (1) | 4.0275 | 1.1495 | 0.0000 |
| 43 | K15 (1) | 2.9775 | 0.6016 | 7.0115 | 116 | L10 (2) | 4.2688 | 1.2244 | 0.0000 |
| 44 | K15 (2) | 2.8961 | 0.5692 | 6.6114 | 117 | L10 (3) | 4.0364 | 1.2772 | 0.0000 |
| 45 | K15 (3) | 3.0788 | 0.6016 | 6.4908 | 118 | Z1 (1) | 3.6662 | 0.8609 | 3.0114 |
| 46 | K16 (1) | 4.7847 | 0.6170 | 5.4757 | 119 | Z1 (2) | 4.1984 | 0.8273 | 3.0368 |
| 47 | K16 (2) | 5.0257 | 0.6739 | 5.6554 | 120 | Z1 (3) | 5.3353 | 0.9575 | 3.7064 |
| 48 | K16 (3) | 4.8076 | 0.6236 | 5.4851 | 121 | Z2 (1) | 9.5390 | 0.0000 | 0.0000 |
| 49 | K17 (1) | 2.6462 | 0.4097 | 3.3136 | 122 | Z2 (2) | 9.6187 | 0.0000 | 0.0000 |
| 50 | K17 (2) | 3.2479 | 0.3741 | 3.4627 | 123 | Z2 (3) | 9.9355 | 0.0000 | 0.0000 |
| 51 | K17 (3) | 3.2578 | 0.3692 | 3.5462 | 124 | Z3 (1) | 8.0185 | 0.8214 | 8.4913 |
| 52 | K18 (1) | 2.8267 | 0.6429 | 7.4100 | 125 | Z3 (2) | 7.7125 | 0.8770 | 8.5008 |
| 53 | K18 (2) | 2.9152 | 0.6307 | 6.8752 | 126 | Z3 (3) | 8.0653 | 0.8415 | 8.2959 |
| 54 | K18 (3) | 3.0225 | 0.5963 | 7.3105 | 127 | Z4 (1) | 1.8157 | 0.0000 | 0.5712 |
| 55 | K19 (1) | 4.5547 | 2.1049 | 4.0637 | 128 | Z4 (2) | 1.8304 | 0.0000 | 0.5468 |
| 56 | K19 (2) | 4.4045 | 2.2414 | 4.1377 | 129 | Z4 (3) | 1.8990 | 0.0000 | 0.6764 |
| 57 | K19 (3) | 4.4148 | 2.1346 | 4.2512 | 130 | Z5 (1) | 2.3105 | 0.3902 | 0.0000 |
| 58 | K20 (1) | 4.2623 | 1.5664 | 4.5670 | 131 | Z5 (2) | 2.2044 | 0.3790 | 0.0000 |
| 59 | K20 (2) | 4.0186 | 1.4907 | 4.3673 | 132 | Z5 (3) | 2.2055 | 0.4133 | 0.0000 |
| 60 | K20 (3) | 3.8155 | 1.5179 | 4.2680 | 133 | Z6 (1) | 0.0000 | 0.0000 | 0.0000 |
| 61 | K21 (1) | 3.5716 | 0.9766 | 7.5713 | 134 | Z6 (2) | 0.0000 | 0.0000 | 0.0000 |
| 62 | K21 (2) | 3.7960 | 1.0256 | 6.9606 | 135 | Z6 (3) | 0.0000 | 0.5506 | 0.0000 |
| 63 | K21 (3) | 3.8024 | 0.9509 | 7.2278 | 136 | Z7 (1) | 0.0000 | 0.8748 | 0.0000 |
| 64 | K22 (1) | 4.1592 | 0.7405 | 7.9692 | 137 | Z7 (2) | 0.0000 | 0.9802 | 0.0000 |
| 65 | K22 (2) | 4.5366 | 0.7796 | 8.7640 | 138 | Z7 (3) | 0.0000 | 1.2859 | 0.0000 |
| 66 | K22 (3) | 4.1205 | 0.8094 | 8.8637 | 139 | Z8 (1) | 0.0000 | 0.3875 | 2.3738 |
| 67 | K23 (1) | 4.4696 | 1.0655 | 3.6310 | 140 | Z8 (2) | 0.0000 | 1.2109 | 0.5796 |
| 68 | K23 (2) | 4.9758 | 1.0639 | 3.8116 | 141 | Z8 (3) | 0.0000 | 1.1256 | 0.6195 |
| 69 | K23 (3) | 4.9040 | 1.1470 | 3.7595 | 142 | Z9 (1) | 0.0000 | 0.5757 | 0.0000 |
| 70 | K24 (1) | 6.5000 | 1.8760 | 5.1748 | 143 | Z9 (2) | 3.4756 | 0.7275 | 1.6793 |
| 71 | K24 (2) | 6.8651 | 1.8775 | 5.2187 | 144 | Z9 (3) | 3.9751 | 0.7648 | 1.7184 |
| 72 | K24 (3) | 6.6490 | 1.7018 | 5.5586 | 145 | Z10 (1) | 1.6561 | 0.3708 | 0.5121 |
| 73 | K25 (1) | 10.0070 | 1.7481 | 2.5969 | 146 | Z10 (2) | 2.3359 | 0.4381 | 0.5501 |
|  |  |  |  |  | 147 | Z10 (3) | 2.1299 | 0.4149 | 0.4761 |

**Table S12** Performances of backpropagation neural network (BPNN) models with different pre-processing for predicting index constituents of *L. aggregata*.

| Constituents | Method | Calibration | | Prediction | | |
| --- | --- | --- | --- | --- | --- | --- |
|  |  | $R_{c}^{2}$ | RMSEC | $R_{p}^{2}$ | RMSEP | RPD |
| Norisoboldine | Raw | 0.7616 | 0.2454 | 0.7183 | 0.3106 | 1.8840 |
|  | SG | 0.7960 | 0.2176 | 0.7815 | 0.2789 | 2.1394 |
|  | SNV | **0.8891** | **0.1614** | **0.8841** | **0.2013** | **2.9369** |
|  | 1^st^D | 0.7410 | 0.2482 | 0.6879 | 0.3292 | 1.7901 |
|  | 2^nd^D | 0.8684 | 0.1740 | 0.8305 | 0.2486 | 2.4289 |
|  | 1^st^D-SG | 0.8384 | 0.1929 | 0.8420 | 0.2400 | 2.5160 |
|  | 2^nd^D-SG | 0.8405 | 0.1915 | 0.7182 | 0.3205 | 1.8839 |
| Linderane | Raw | 0.8109 | 0.0638 | 0.6488 | 0.0870 | 1.6873 |
|  | SG | 0.7005 | 0.0809 | 0.5442 | 0.0976 | 1.4811 |
|  | SNV | **0.8620** | **0.0538** | **0.8798** | **0.0526** | **2.8838** |
|  | 1^st^D | 0.8211 | 0.0609 | 0.7590 | 0.0760 | 2.0371 |
|  | 2^nd^D | 0.6836 | 0.0790 | 0.6422 | 0.0968 | 1.6717 |
|  | 1^st^D-SG | 0.8019 | 0.0632 | 0.7926 | 0.0720 | 2.1960 |
|  | 2^nd^D-SG | 0.8500 | 0.0542 | 0.8537 | 0.0621 | 2.6141 |
| Lindenenol | Raw | 0.8204 | 0.2138 | 0.7835 | 0.2926 | 2.1493 |
|  | SG | 0.8014 | 0.2328 | 0.8094 | 0.2747 | 2.2904 |
|  | SNV | **0.8881** | **0.1793** | **0.8800** | **0.1950** | **2.8865** |
|  | 1^st^D | 0.7737 | 0.2530 | 0.7612 | 0.2828 | 2.0463 |
|  | 2^nd^D | 0.7264 | 0.2744 | 0.5519 | 0.3114 | 1.4938 |
|  | 1^st^D-SG | 0.8167 | 0.2218 | 0.7925 | 0.2783 | 2.1951 |
|  | 2^nd^D-SG | 0.8691 | 0.1871 | 0.8261 | 0.2558 | 2.3977 |

$R_{c}^{2}$: coefficients of determination for calibration; RMSEC: root mean square error of calibration; $R_{P}^{2}$: coefficients of determination for prediction; RMSEP: root mean square error of prediction; RPD: ratio of prediction to deviation; SG; Savitzky-Golay; SNV: standard normal variate; 1^st^D: first-order derivativ; 2^nd^D: second derivative.

**
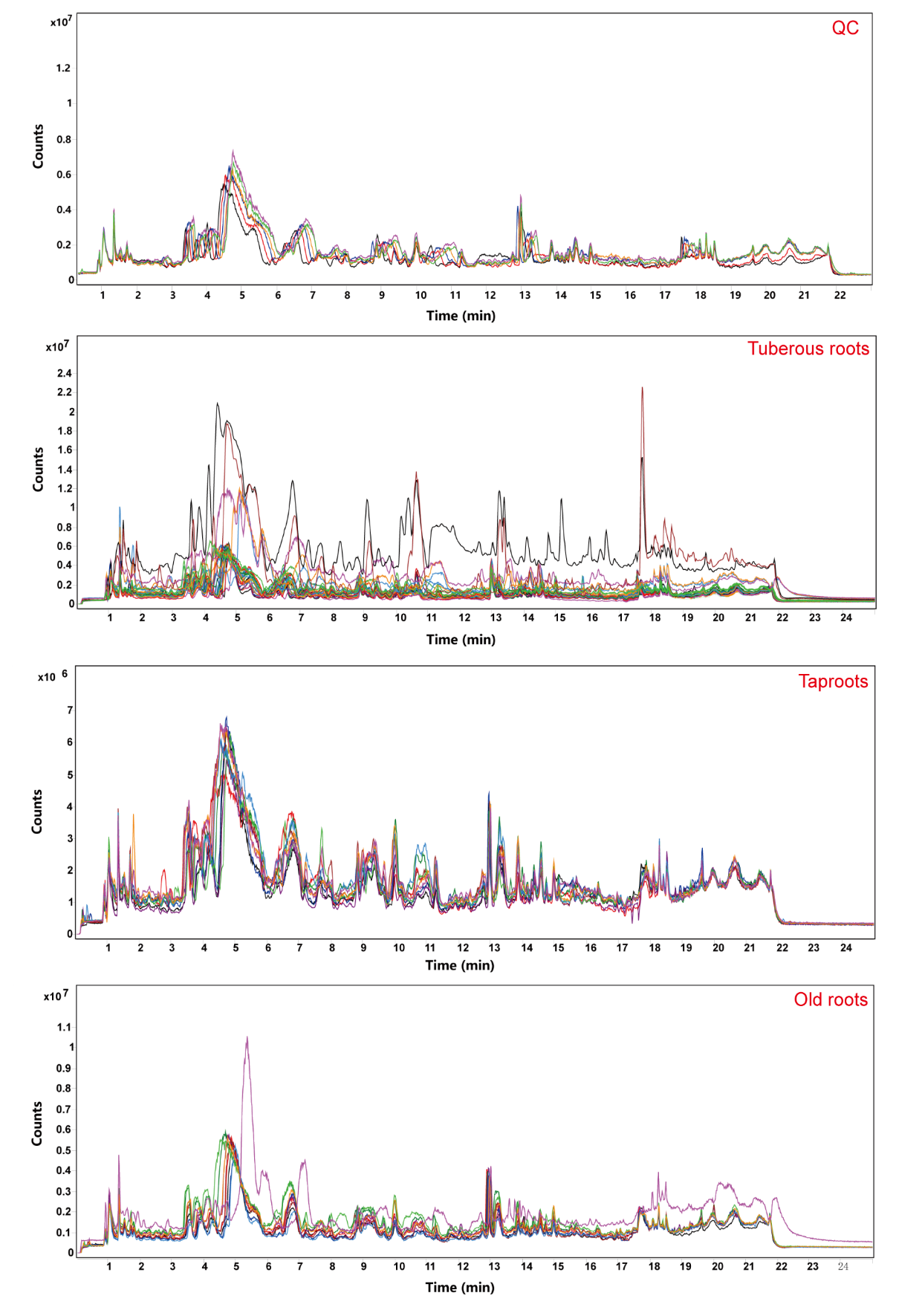
**

**Fig. S1** The total ion current (TIC) overlapping map in positive ion mode by ultra-performance liquid chromatography-quadrupole time-of-flight mass spectrometry (UPLC-QTOF-MS) of three root types of *L. aggregata* samples, including quality control (QC) sample results. Note: The abscissa is the acquisition time of the metabolite. The ordinate is the ion current intensity of the ion detection. QC: quality control.


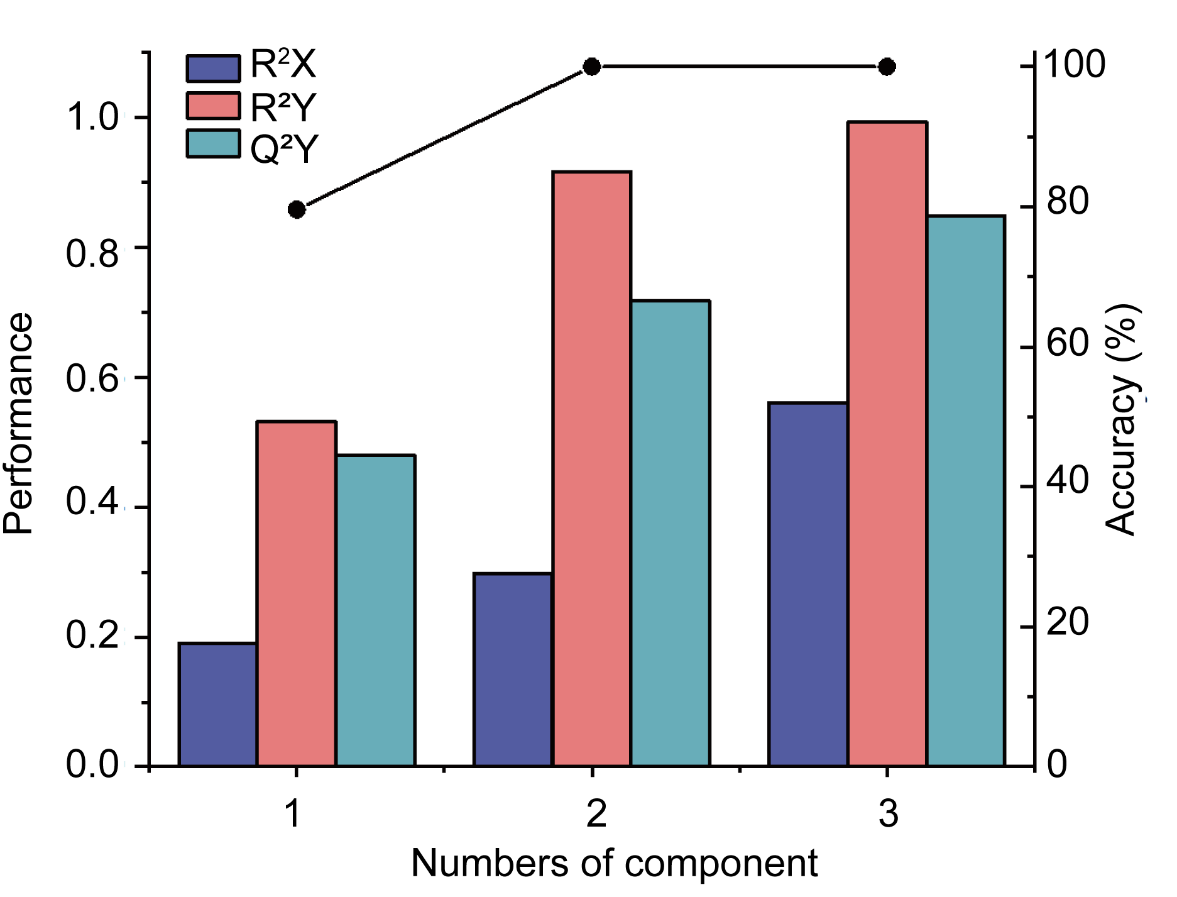


**Fig. S2** Cross-validation based on the corresponding (partial least squares discrimination analysis) PLS-DA model based on ultra-performance liquid chromatography-quadrupole time-of-flight mass spectrometry (UPLC-QTOF-MS) metabolomics data. The model’s predictive power can be reflected in the magnitude of the Q^2^ value, while R^2^ represents the total variance the model can explain.


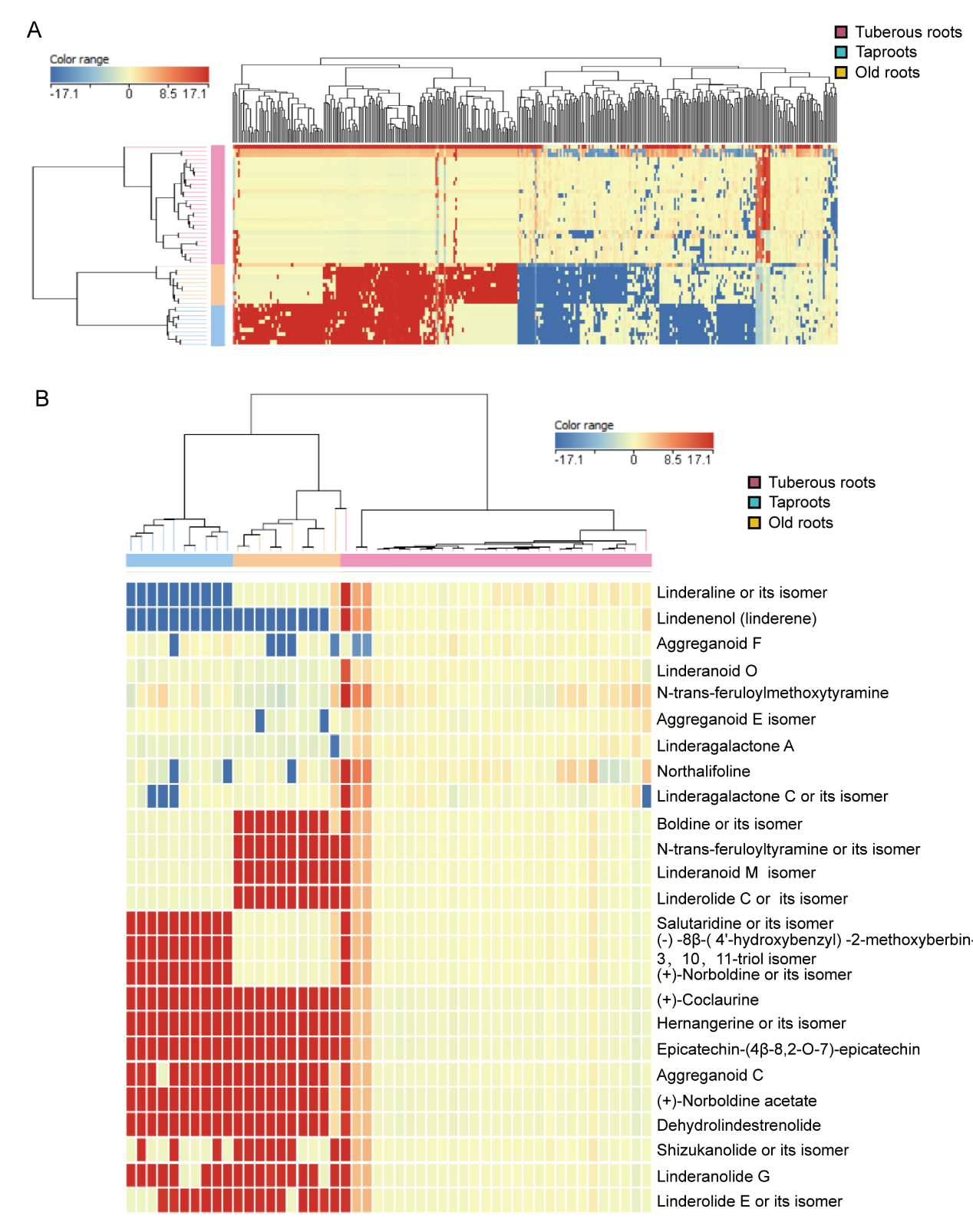


**Fig. S3** Heatmaps of hierarchical cluster analysis (HCA) by ultra-performance liquid chromatography-quadrupole time-of-flight mass spectrometry (UPLC-QTOF-MS) in three root types of *L. aggregata*. (A) The heatmap of HCA of total differential metabolites. (B) The heatmap of HCA of 25 differential metabolites.


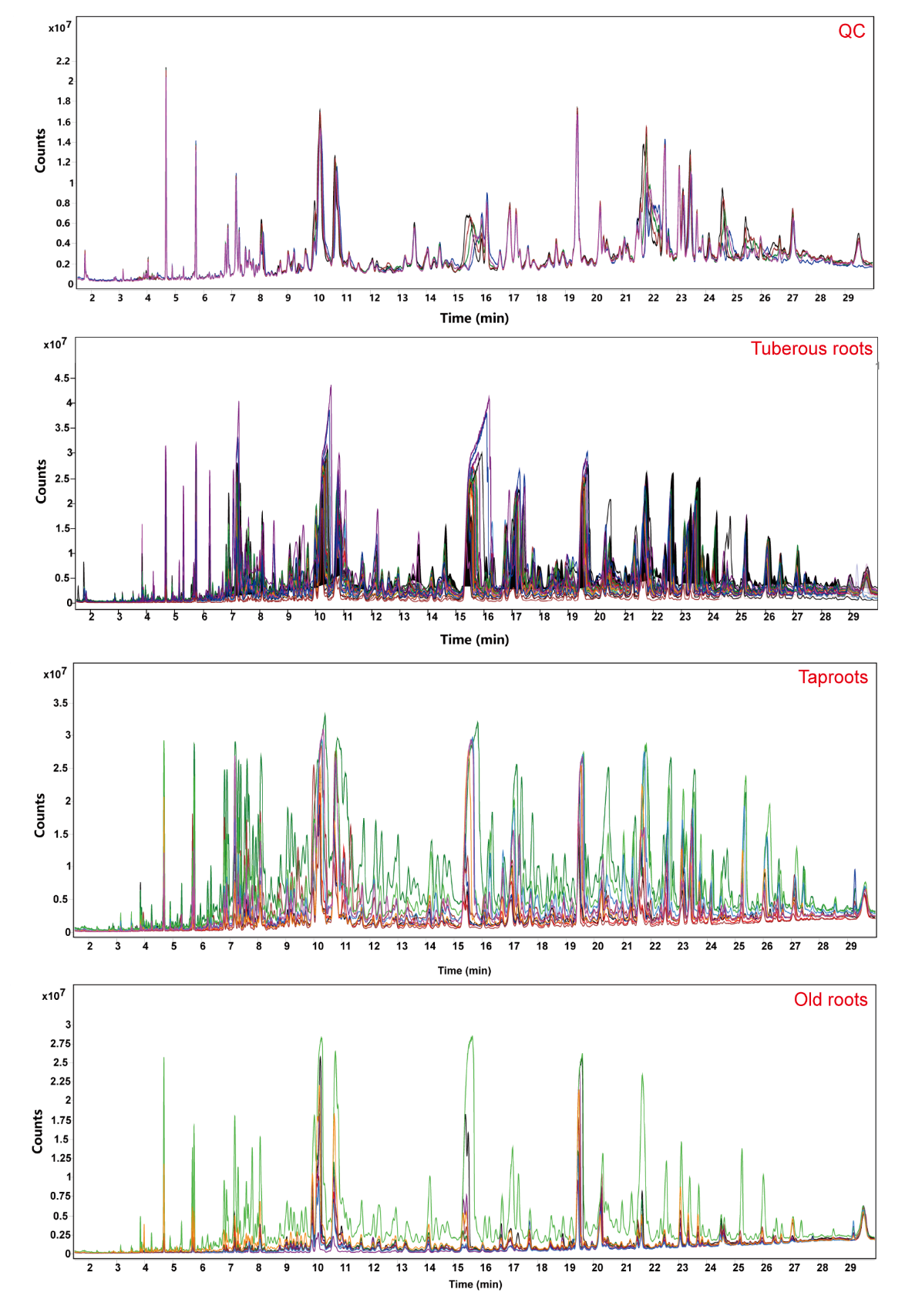


**Fig. S4** The total ion current (TIC) overlapping map in positive ion mode by gas chromatography-mass spectrometry (GC-MS) of three root types of *L. aggregata* samples, including quality control (QC) sample results. Note: The abscissa is the acquisition time of the metabolite. The ordinate is the ion current intensity of the ion detection. QC: quality control.


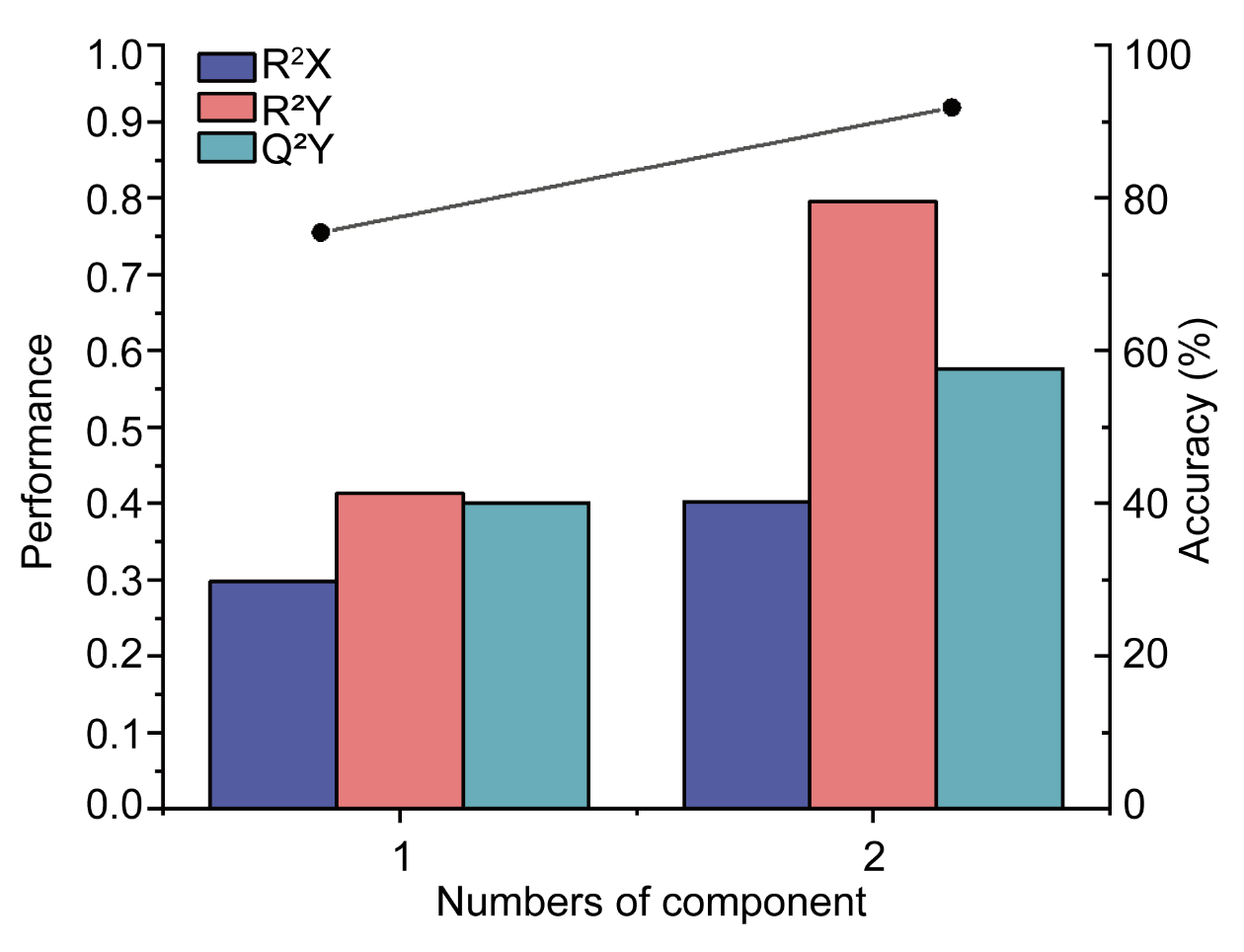


**Fig. S5** Cross-validation based on the corresponding (partial least squares discrimination analysis) PLS-DA model based on gas chromatography-mass spectrometry (GC-MS) metabolomics data. The model’s predictive power can be reflected in the magnitude of the Q^2^ value, while R^2^ represents the total variance the model can explain.


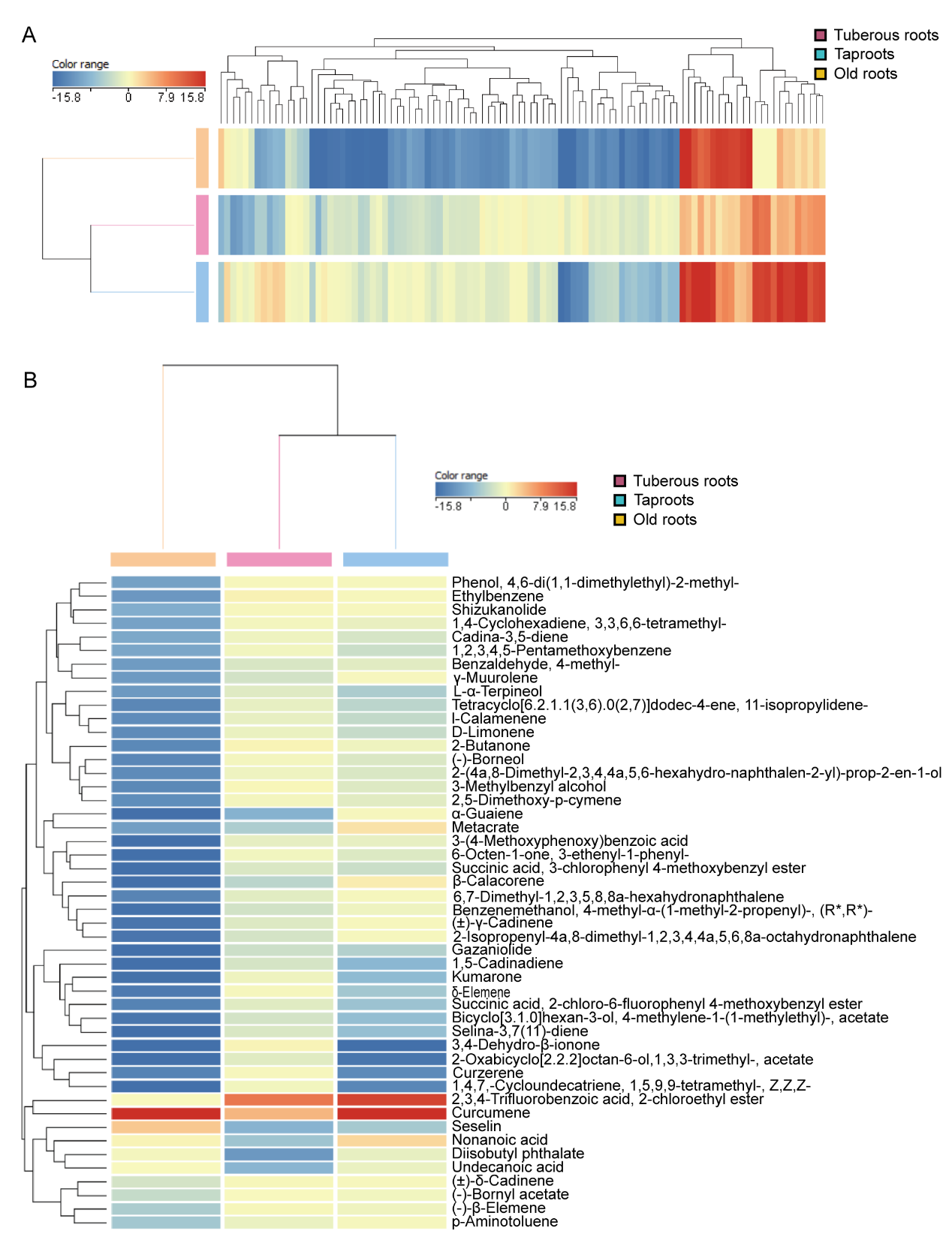


**Fig. S6** Heatmaps of hierarchical cluster analysis (HCA) by gas chromatography-mass spectrometry (GC-MS) in three root types of *L. aggregata*. (A) The heatmap of HCA of total differential metabolites. (B) The heatmap of HCA of 48 differential metabolites.


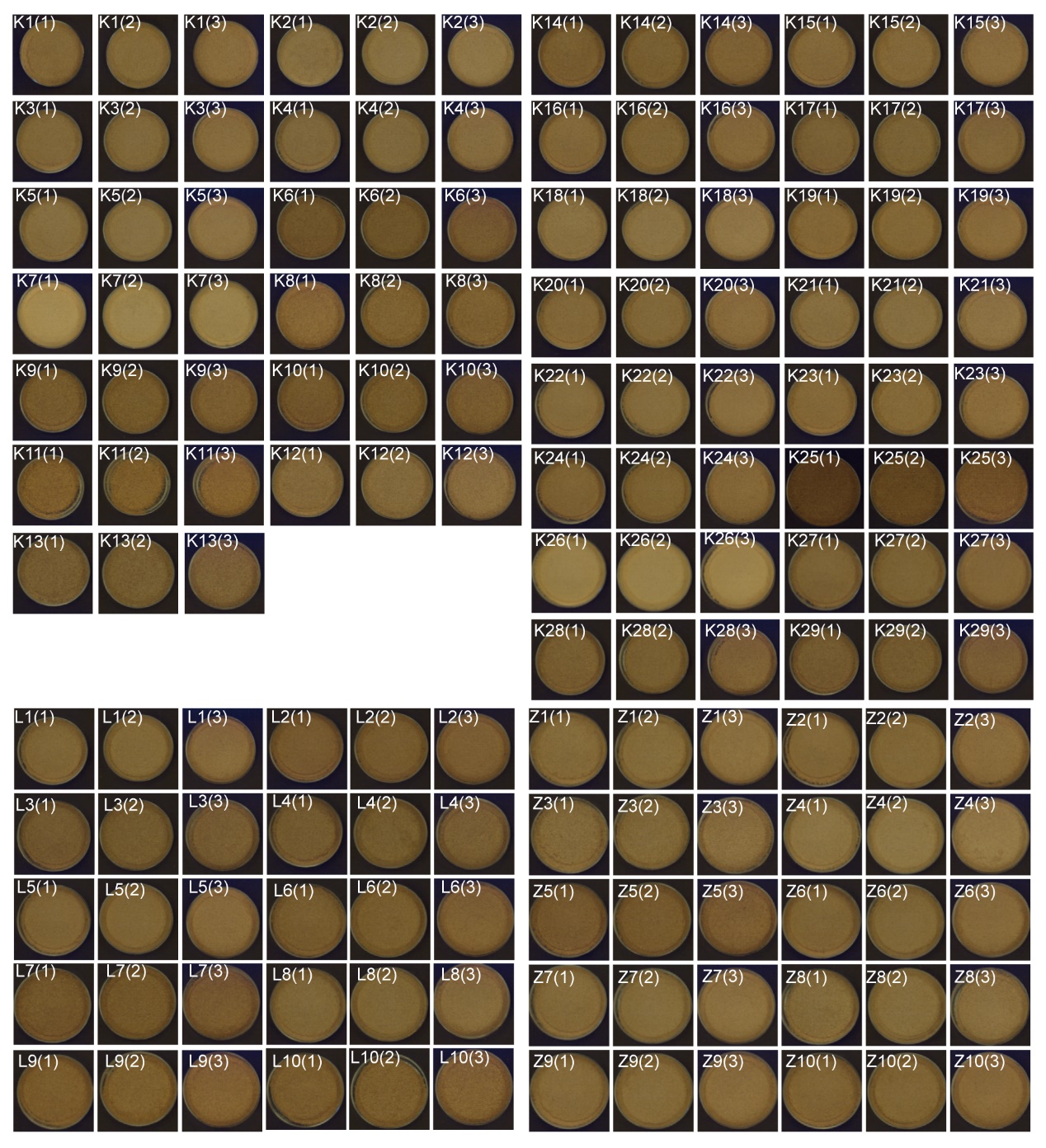


**Fig.****S7** Hyperspectral images of all samples.
